
JOURNAL INFORMATION
==============================
NLM Title Abbreviation: Eur Psychiatry
Journal ID: Eur Psychiatry
NLM Journal ID: 9111820
Journal ID: EPA

European Psychiatry

ISSN: 0924-9338
EISSN: 1778-3585
Publisher: Cambridge University Press

ARTICLE INFORMATION
==============================
PMCID: PMC9412732
DOI: 10.1192/j.eurpsy.2020.95
Article ID: S0924933820000954
Article version: 1
Subjects: Abstracts

EPA 2020 Abstracts, Part 2

Publication date: 2020 Jul
Electronic publication date: 2020 Dec 8
Volume: 63
Issue: Suppl 1
Issue title: Abstracts of the 28th European Congress of Psychiatry
First page: S629
Last page: S712
Copyright: © The Author(s) 2020
Copyright year: 2020
Copyright holder: The Author(s)
License: This is an Open Access article, distributed under the terms of the Creative Commons Attribution licence (http://creativecommons.org/licenses/by/4.0/), which permits unrestricted re-use, distribution, and reproduction in any medium, provided the original work is properly cited.
License URL: https://creativecommons.org/licenses/by/4.0/

Erratum in: EPA 2020 Abstracts, Part 2 - ADDENDUM 63 Suppl 1 8 12 2020 S713 S713 European Psychiatry PMC9412711
Erratum in: EPV0798 - CORRIGENDUM 63 Suppl 1 4 9 2020 S714 S714 European Psychiatry PMC9412719
Figure count: 9
Table count: 5
Page count: 84

==============================

Article ID: O0050
Subjects: Abstract, Oral Communication

Construct and concurrent validity of illness and help-seeking behaviour scale (IHSBS) in portuguese mental health patients

Macedo A.
Silva C. *
Soares M.J.
Araújo A.
Pereira A.T.
Madeira N.
Moura D.
Coroa M.
Morais S.
Rosendo I.
Miranda A.F.
Portugal
* Corresponding author.
Keywords: IHSBS, Stigma, mental health

Conflict of interest:

No

Article ID: O0058

Neurotoxic effects of varying doses of tramadol on the cerebral cortex of adult male wistar rats

Adebisi B. *
Nigeria
* Corresponding author.
Keywords: Cerebral cortex, Neurotoxic, tramadol, Neuronal dysfunction

Conflict of interest:

No

Article ID: O0080

Associations between thyroid hormone levels and clinical outcomes in schizophrenic inpatients

Jung M.J. *
Lee S.H.
Lee T.K.
Choi J.H.
Korea , Republic of
* Corresponding author.
Keywords: Thyroid hormones, schizophrenia, Clinical outcomes, social security disability

Conflict of interest:

No

Article ID: O0087

Analysis of microrna expressions in schizophrenia: Insights into molecular interactions and functional associations underpinning neurodevelopmental processes

Chen B.-Y. *
Lin J.-J.
Lu M.-K.
Lin S.-H.
Taiwan
* Corresponding author.
Keywords: schizophrenia, microRNA, neurodevelopment, non-psychotic first-degree relatives, bipolar disorder

Conflict of interest:

No

Article ID: O0108

The relation between changes in vitamin D and vitamin B12 levels, treatment characteristics, and outcome in methadone maintenance treatment patients

Malik E. *
Rozner L.
Adelson M.
Schreiber S.
Peles E.
Israel
* Corresponding author.
Keywords: Methadone maintenance treatment, Body mass index (BMI), Vitamin D, Vitamin B12

Conflict of interest:

No

Article ID: O0118

Gender differences in body size perception, dieting and mental distress amongst adolescents, the nord-trøndelag health study (YH3)

Sardahaee F. *
Holmen T.
Kvaløy K.
Norway
* Corresponding author.

Conflict of interest:

No

Article ID: O0129

Major depressive disorder is associated to increased levels of proinflammatory cytokines and alterations in monocytes

Alvarez-Mon M. *
Gomez A.
Orozco A.
Lahera G.
Sosa M.D.
Auba E.
Albillos A.
Alvarez-Mon M.
Spain
* Corresponding author.
Keywords: major depressive disorder, Monocytes, cytokines, systemic inflammation

Conflict of interest:

No

Article ID: EPP0001
Subjects: Abstract, E-Poster Presentations Addictive disorders - Part I

Prevalence of substance use disorder in asperger’s – a case report and review of literature

Ahmad Z. 1 *
Gunturu S. 2
Shengelia R. 3
Zamiri A. 3
Korenis O. 4
1 BronxCare Hospital Center , Psychiatry , Bronx , United States of America
2 BronxCare Hoospital , Psychiatry , Bronx , United States of America
3 BronxCare Health System , Psychiatry , Bronx , United States of America
4 BronxCare, Psychiatry , Bronx , United States of America
* Corresponding author.
Keywords: substance use disorder, Autism Spectrum Disorder, dual mental and substance use disorder, Asperger syndrome

Conflict of interest:

No

Article ID: EPP0034
Subjects: Abstract, Addictive disorders - Part III

Relapse after alcohol detoxification in an acute psychiatric ward: a tertiary care center based cross-sectional study

Gonçalves Cerejeira J. *
Santos Carrasco I.D.L.M.
Capella C.
Rodríguez E.
De Lorenzo M.
De Uribe N.
Gómez M.
Queipo De Llano M.
Gonzaga A.
Guerra G.
Santiago O.
Hospital Clinic of Valladolid , Psychiatry, Valladolid, Spain
* Corresponding author.
Keywords: relapse, alcohol use disorder

Conflict of interest:

No

Article ID: EPP0067
Subjects: Abstract, Addictive disorders - Part VII

Complications in chronic alcoholism: extrapontine myelinolysis

Torralba Viorreta R. 1 *
Gomez-Peinado A. 2
Quero-Palomino V. 1
Martínez Larumbe S. 3
1 Hospital General Universitario de Ciudad Real , Psychiatry, Ciudad Real , Spain
2 Hospital General Universitario de Ciudad Real , Psiquiatría, Ciudad Real , Spain
3 Hospital Universitario Ramón y Cajal , Psychiatry, Madrid , Spain
* Corresponding author.
Keywords: hyponatremia, extrapontine myelinolysis, alcohol use disorder

Conflict of interest:

No

Article ID: EPP0076

Screening for alcohol-related cognitive impairments – a case study

Gil L. 1
Lázaro M. 2
Ferreira S. 3
Diegues P. 3
Teixeira J. 3 *
Mota T. 3
1 Centro Hospitalar Psiquiátrico de Lisboa , Clínica 6, Lisbon , Portugal
2 Centro Hospitalar Psiquiátrico de Lisboa , Clínica 5, Lisboa , Portugal
3 Centro Hospitalar Psiquiátrico de Lisboa , Serviço Alcoologia E Novas Dependências, Lisboa , Portugal
* Corresponding author.
Keywords: Alcohol dependence, Cognitive impairment, remediation, relapse

Conflict of interest:

No

Article ID: EPP0095
Subjects: Abstract, Anxiety disorders and somatoform disorders - Part II

Migraine comorbidity with anxiety disorder, a study in regional hospital durres, Albania

Shemsi (Harizi) E. 1 *
Shemsi K. 2
Domi F. 3
1 Regional Hospital Durres , Neurology , Quarttier Street A.Goga, Albania
2 University of Medicine Tirana , Faculty Of Medicine, Tirana , Albania
3 Regional Hospital Durres , Emergency, Durres , Albania
* Corresponding author.
Keywords: Migraine, Comorbidity, Anxiety, Disorder

Conflict of interest:

No

Article ID: EPP0102
Subjects: Abstract, Bipolar disorders - Part I

Impact of childhood trauma in patients with bipolar disorder.

Betriu M. *
Guàrdia Delgado A.
Navarra G.
Serra-Blasco M.
Massons C.
Crivillés S.
Guinovart M.
Monreal J.A.
Palao Vidal D.
Cardoner Ávarez N.
Parc Taulí University Hospital. Autonomous University of Barcelona (UAB) . I3PT. CIBERSAM, Department of Mental Health, Sabadell , Spain
* Corresponding author.
Keywords: Bipolar disorder, cognitive performance, childhood trauma, clinical features

Conflict of interest:

No

Article ID: EPP0103

Gut feeling: the connection between gut microbiota and bipolar affective disorder

Brás J. *
Sousa R.
Guedes B.
Costa A.
Costa A.
Centro Hospitalar Tondela-Viseu , Department of Psychiatry and Mental Health, VISEU , Portugal
* Corresponding author.
Keywords: Bipolar disorder, gut microbiota, gut-brain axis, Inflammation

Conflict of interest:

No

Article ID: EPP0104

A clinical overview of bipolar disorder among voluntary and involuntary admitted patients in a psychiatric service during 2018: clinical differences and similarities

Caldas F. 1 *
Frias P. 1
Valido R. 1
Barros M. 1
Pires D. 1
Gonçalves M. 2
Falcão M. 3
1 Hospital de Magalhães Lemos , Psychiatry, Porto , Portugal
2 Hospital de Magalhães Lemos , Psychiatry, porto , Portugal
3 Centro Hospitalar do Porto, Centro Materno Infantil Do Norte , porto , Portugal
* Corresponding author.
Keywords: compulsory hospitalization, Bipolar disorder, voluntary hospitalisation

Conflict of interest:

No

Article ID: EPP0109
Subjects: Abstract, Bipolar disorders - Part II

Gender differences among admitted patients with bipolar disorder during 2018

Caldas F. 1 *
Frias P. 1
Valido R. 1
Gonçalves M. 2
Pires D. 1
Barros M. 1
Falcão M. 3
1 Hospital de Magalhães Lemos , Psychiatry, Porto , Portugal
2 Hospital de Magalhães Lemos , Psychiatry, porto , Portugal
3 Centro Hospitalar do Porto, Centro Materno Infantil Do Norte , porto , Portugal
* Corresponding author.
Keywords: Bipolar disorder, Gender

Conflict of interest:

No

Article ID: EPP0123
Subjects: Abstract, Bipolar disorders - Part III

Metabolic disturbances as prognostic factors in bipolar disorder: a systematic review.

Giménez Palomo A. 1 *
Dodd S. 2
Berk M. 2
Gomes S.P. 1
Pons M.T. 3
Anmella G. 4
Pinzon Espinosa J. 5
Pacchiarotti I. 6
1 Hospital Clínic , Psychiatry, Barcelona , Spain
2 Barwon Health, Impact Src , Geelong , Australia
3 Hospital Clinic de Barcelona , Psychiatry, Barcelona , Spain
4 Hospital Clinic, University of Barcelona, IDIBAPS, CIBERSAM , Schizophrenia Unit , Institute Of Neuroscience , Barcelona , Spain
5 Hospital Clinic de Barcelona, Institut Clinic De Neurociencias , Barcelona , Spain
6 Bipolar and Depressive Disorders Unit, Hospital Clínic de Barcelona , Psychiatry, Barcelona , Spain
* Corresponding author.
Keywords: Bipolar disorder, course of illness, prognosis, metabolic syndrome

Conflict of interest:

No

Article ID: EPP0145
Subjects: Abstract, Bipolar disorders - Part V

International convergences and divergences in meta-analyses of epidemiological studies of bipolar disorder

Moreira A.L. R. 1 *
Youngstrom E. 2
Van Meter A. 3
1 Centro Hospitalar do Oeste, Departamento De Psiquiatria E Saúde Mental, Caldas da Rainha , Portugal
2 University of North Carolina at Chapel Hill , Department of Psychology and Neuroscience, Chapel Hill , NC , United States of America
3 The Donald and Barbara Zucker School of Medicine at Hofstra/Northwell, Department of Psychiatry, New York , United States of America
* Corresponding author.
Keywords: epidemiology, community, Bipolar disorder, International

Conflict of interest:

No

Article ID: EPP0157
Subjects: Abstract, Child and adolescent psychiatry - Part I

Autistic traits among Saudi university students; relations with emotional intelligence and alexithymia

Soliman E.S. 1
Almutairi A. 2 *
Almutairi F. 2
Alswailem H. 2
Salem R. 2
Albati W. 2
1 College of Medicine, Zagazig University , Psychiatry Department, Zagazig , Egypt
2 Princess Nourah Bint Abdulrahman University , College Of Medicine, Riyadh , Saudi Arabia
* Corresponding author.
Keywords: Autism spectrum disease (ASD), Alexithymia, emotional intelligence, Saudi university students

Conflict of interest:

No

Article ID: EPP0161

Long acting injectable antipsychotics in children and adolescents with serious mental illness

Alvites Ahumada M.D.P. 1 *
Landa E. 2
Serrano N. 2
Guijarro S. 2
Díaz Fernandez M.D.C. 2
Infante Sanchez De Lugarnuevo M. 2
1 Complejo Hospitalario Mancha Centro, Unidad De Salud Mental Infanto Juvenil, Alcázar de San Juan (Ciudad Real) , Spain
2 Hospital general de Tomelloso, Unidad De Salud Mental , Tomelloso , Spain
* Corresponding author.
Keywords: long acting injectable antipsychotics, Children, adolescents, serious mental illness

Conflict of interest:

No

Article ID: EPP0193
Subjects: Abstract, Child and adolescent psychiatry - Part IV

From children pathology to psychosis. trauma and substancie use as risk marks.

Garcia E. *
Garcia M.
Barredo P.
Sánchez Cabezudo A.
Soto M.
Sescam , Psychiatry, Toledo , Spain
* Corresponding author.
Keywords: trauma, psychosis, Children, adolescence

Conflict of interest:

No

Article ID: EPP0207
Subjects: Abstract, Child and adolescent psychiatry - Part V

Acceptability and utility of psychoeducation and cognitive behavioural therapy for parents of children with functional neurological disorders: a pilot study

Baig B. 1
Bhatt H. 1
Gómez-Vallejo S. 2 *
Cooney M. 1
Kuruppuarachchi N. 1
1 Kings College London , Child and Adolescent Psychiatry, Ioppn, London , United Kingdom
2 King's College London , Department of Child & Adolescent Psychiatry, London , United Kingdom
* Corresponding author.
Keywords: functional neurological disorders, Medically unexplained symptoms, family therapy, CBT

Conflict of interest:

No

Article ID: EPP0257
Subjects: Abstract, Child and adolescent psychiatry - Part X

“How to understand the development of identity and gender diversity in adolescents and young adults with autistic spectrum disorder (ASD)”

Vandeusen T.
Yale University School of Medicine , Psychiatry, New Haven , United States of America
Keywords: child and adolescent development, gender identity, gender dysphoria, autistic spectrum disorder

Conflict of interest:

No

Article ID: EPP0268
Subjects: Abstract, Classification of Mental Disorders

Social phobia and autism: two peas in a pod?

Caetano F. 1 *
Araújo M. 2
Samouco A. 3
Priscila C. 1
Frias P. 4
Carvalho S. 1
1 Hospital de Magalhães Lemos , Serviço De Internamento B, Porto , Portugal
2 Hospital de Magalhães Lemos , Serviço De Internamento C, Porto , Portugal
3 Unidade Local de Saúde do Norte Alentejano , Department of Psychiatry and Mental Health, Portalegre , Portugal
4 Hospital de Magalhães Lemos , Psychiatry, Porto , Portugal
* Corresponding author.
Keywords: SOCIAL PHOBIA, Autism

Conflict of interest:

No

Article ID: EPP0272

Major depression as an expression of frontal lobe dysfunction

Valido R. *
Caldas F.
Barros M.
Ferreira P.
Hospital de Magalhães Lemos , Psychiatry, Porto , Portugal
* Corresponding author.
Keywords: Frontal Lobe, Prefrontal syndrom, Major Depressive Episode, Executive Cognitive Functions

Conflict of interest:

No

Article ID: EPP0281
Subjects: Abstract, Comorbidity / dual pathologies - Part I

Dual pathology and gender differences

Caldas F. 1 *
Frias P. 2
Valido R. 2
Barros M. 2
Pires D. 2
Gonçalves M. 1
Falcão M. 3
1 Hospital de Magalhães Lemos , Psychiatry, porto , Portugal
2 Hospital de Magalhães Lemos , Psychiatry, Porto , Portugal
3 Centro Hospitalar do Porto, Centro Materno Infantil Do Norte , porto , Portugal
* Corresponding author.
Keywords: Gender, dual pathology

Conflict of interest:

No

Article ID: EPP0291
Subjects: Abstract, Comorbidity / dual pathologies - Part II

Interictal dysphoric disorder in patients with and without epilepsy

Kustov G. 1 *
Rider F. 2
Pashnin E. 3
Pochigaeva K. 4
Yakovlev A. 3
Zinchuk M. 5
1 Moscow Research and Clinical Center for Neuropsychiatry, Department of New Methods Of Therapy Of Mental Disorders, Moscow , Russian Federation
2 Moscow Research and Clinical Center for Neuropsychiatry, Department of Epidemiology- Prophylaxis and Healthcare, Moscow , Russian Federation
3 Moscow Research and Clinical Center for Neuropsychiatry, Suicidology (crisis), Moscow , Russian Federation
4 Moscow Research and Clinical Center for Neuropsychiatry, Department of Nonpsychotic Mental Disorders In Neurologic and Geriatric Patients, Moscow , Russian Federation
5 Moscow research and clinical center for neuropsychiatry, Suicidology (crisis), Moscow , Russian Federation
* Corresponding author.
Keywords: Interictal dysphoric disorder, major depressive disorder, Dépression, Epilepsy

Conflict of interest:

No

Article ID: EPP0292

Comorbidity of bipolar affective disorder and epilepsy

Kustov G. 1 *
Pashnin E. 2
Zinchuk M. 3
Akzhigitov R. 4
1 Moscow Research and Clinical Center for Neuropsychiatry, Department of New Methods Of Therapy Of Mental Disorders, Moscow , Russian Federation
2 Moscow Research and Clinical Center for Neuropsychiatry, Suicidology (crisis), Moscow , Russian Federation
3 Moscow research and clinical center for neuropsychiatry, Suicidology (crisis), Moscow , Russian Federation
4 Moscow Research and Clinical Center for Neuropsychiatry, Deputy Director, Moscow , Russian Federation
* Corresponding author.
Keywords: Epilepsy, Bipolar disorder, Bipolar depression

Conflict of interest:

No

Article ID: EPP0310
Subjects: Abstract, Comorbidity / dual pathologies - Part IV

“Drink to forget” – a case report

Lázaro M. 1
Gil L. 2
Ferreira S. 3
Diegues P. 3
Teixeira J. 3 *
Mota T. 3
1 Centro Hospitalar Psiquiátrico de Lisboa , Clínica 5, Lisboa , Portugal
2 Centro Hospitalar Psiquiátrico de Lisboa , Clínica 6, Lisboa , Portugal
3 Centro Hospitalar Psiquiátrico de Lisboa , Serviço Alcoologia E Novas Dependências, Lisboa , Portugal
* Corresponding author.
Keywords: Alcohol dependence, Cognitive impairment, Anxiety, relapse

Conflict of interest:

No

Article ID: EPP0323
Subjects: Abstract, Consultation liaison psychiatry and psychosomatics - Part I

CPAP-induced mania in a man with obstructive sleep apnea

Esteves-Sousa D. 1 *
Facucho-Oliveira J. 2
Albuquerque M. 1
Moura N. 3
Espada-Santos P. 1
Mendonça L. 1
1 Hospital de Cascais Dr. José de Almeida , Departamento De Psiquiatria E Saúde Mental, Alcabideche , Portugal
2 Hospital de Cascais , Department of Psychiatry, Cascais , Portugal
3 Centro Hospitalar Lisboa Ocidental , Department of Psychiatry, Lisboa , Portugal
* Corresponding author.
Keywords: CPAP, Bipolar disorder, OBSTRUCTIVE SLEEP APNEA, mania

Conflict of interest:

No

Article ID: EPP0369
Subjects: Abstract, Depressive disorders - Part I

Examining the effectiveness of a culturally appropriate intervention in reducing postpartum depression among bedouin women in southern israel

Alfayumi-Zeadna S.
Ben-Gurion University , Public Health, Lehavem , Israel
Keywords: postpartum depression, Bedouin women in Israel, culturally appropriate intervention

Conflict of interest:

No

Article ID: EPP0385
Subjects: Abstract, Depressive disorders - Part II

Transcranial direct-current stimulation (tDCS) as an adjuvant therapy for treatment-resistant depression (TRD)

Facucho-Oliveira J. 1 *
Esteves-Sousa D. 1
Espada-Santos P. 1
Albuquerque M. 2
Costa M. 2
Mendonça L. 2
Neves R. 3
1 Hospital de Cascais , Department of Psychiatry, Cascais , Portugal
2 Hospital de Cascais Dr. José de Almeida , Departamento De Psiquiatria E Saúde Mental, Alcabideche , Portugal
3 Casa de Saúde da Idanha, Irmãs Hospitaleiras do Sagrado Coração de Jesus , Department of Psychiatry, Lisboa , Portugal
* Corresponding author.
Keywords: tDCS, Depression, TRD

Conflict of interest:

No

Article ID: EPP0390

Improvement in depressive symptoms and various functional aspects after treatment of major depression with vortioxetine. Interim results from the “real-life effectiveness of vortioxetine” (relieve) study

Llorca P.-M. 1
Jain R. 2
Touya M. 3
Simonsen K. 4
Hammer-Helmich L. 5 *
1 University Clermont Auvergne , Department of Psychiatry, Clermont Ferrand , France
2 Texas Tech University School of Medicine , Department of Psychiatry, Lubbock , United States of America
3 Lundbeck, Health Economics and Outcomes Research , Deerfield , United States of America
4 H. Lundbeck A/S, Health Economic and Epidemiology Statistics , Valby , Denmark
5 H. Lundbeck A/S, Medical Affairs Value Evidence , Valby , Denmark
* Corresponding author.
Keywords: vortioxetine, Real World Evidence, functional impairment, Depressive symptoms

Article ID: EPP0441
Subjects: Abstract, Eating disorders - Part I

Anorexia and autism: which spectrum are we speaking about?

Duarte T. 1 *
Fernandes Órfão F. 2
Cordeiro C. 1
Duarte Silva I. 1
Côrte-Real B. 1
Andrade G. 1
Neves A. 1
1 Centro Hospitalar Universitário Lisboa Norte, Serviço De Psiquiatria E Saúde Mental , Lisboa , Portugal
2 ACES Almada Seixal, Usf Cuidar Saúde , Seixal , Portugal
* Corresponding author.
Keywords: Anorexia nervosa, autism spectrum disorders, eating disorders, female ASD

Conflict of interest:

No

Article ID: EPP0508
Subjects: Abstract, Emergency psychiatry - Part I

Practical experiences with agitation in psychiatric emergencies (practice): design and rationale

Kahl K. 1 *
Messer T. 2
Bradl S. 2
Kuhn J. 3
1 Hannover Medical School , Psychiatry, Social Psychiatry and Psychotherapy , Hannover , Germany
2 Danuvius Klinik Pfaffenhofen , Psychiatry, Pfaffenhofen a.d. Ilm, Germany
3 Evangelisches Krankenhaus Niederrhein , Psychiatrie, Psychotherapie Und Psychosomatik, Oberhausen, Germany
* Corresponding author.
Keywords: Agitation, PATIENT SATISFACTION, emergency treatment

Article ID: EPP0517
Subjects: Abstract, Epidemiology and Social Psychiatry - Part I

Socio-demographic characterization of the population accompanied by the mental health home-based care team at the vale do sousa region

Barranha R. *
Gomes D.
Ribeiro J.P.
Von Doellinger O.
Centro Hospitalar do Tâmega e Sousa , Department of Psychiatry and Mental Health, Penafiel , Portugal
* Corresponding author.
Keywords: Home-base care team, Severe mental illness, Social support, Socio-demographic characterization

Conflict of interest:

No

Article ID: EPP0520

Prescribing patterns of anxiolytic medication among physician specialties in the slovak republic in 2014-2017

Brazinova A. 1 *
Sivcova V. 1
Breznoscakova D. 2
1 Faculty of Medicine, Comenius University, Institute Of Epidemiology , Bratislava , Slovak Republic
2 PJ Safarik University , Department of Psychiatry, Kosice , Slovak Republic
* Corresponding author.
Keywords: anxiolytic, médication, prescription, Psychiatric

Conflict of interest:

No

Article ID: EPP0564
Subjects: Abstract, Forensic psychiatry - Part I

Involuntarily admitted patients in a psychiatric hospital during 2018

Caldas F. 1 *
Frias P. 1
Valido R. 1
Gonçalves M. 2
Pires D. 1
Barros M. 1
Falcão M. 3
1 Hospital de Magalhães Lemos , Psychiatry, Porto , Portugal
2 Hospital de Magalhães Lemos , Psychiatry, porto , Portugal
3 Centro Hospitalar do Porto, Centro Materno Infantil Do Norte , porto , Portugal
* Corresponding author.
Keywords: involuntary admittion, serious mental illness

Conflict of interest:

No

Article ID: EPP0585
Subjects: Abstract, Genetics & Molecular neurobiology

No association between akt1 gene variants and schizophrenia in the russian population

Poltavskaya E. 1 *
Kornetova E. 2
Boiko A. 3
Fedorenko O. 1
1 Tomsk National Research Medical Center of the Russian Academy of Sciences, Mental Health Research Institute , Tomsk , Russian Federation
2 Mental Health Research Institute , Tomsk National Research Medical Center, Russian Academy of Sciences, Department of Endogenous Disorders, Tomsk , Russian Federation
3 Mental Health Research Institute , Tomsk National Research Medical Center of the Russian Academy of Sciences, Laboratory Of Molecular Genetics and Biochemistry, Tomsk , Russian Federation
* Corresponding author.
Keywords: AKT1, gene, polymorphism, schizophrenia

Conflict of interest:

No

Article ID: EPP0591
Subjects: Abstract, Intellectual Disability

Serotoninergic toxicity triggered by low doses of sertraline in a patient with intellectual disability.

Giménez Palomo A. *
Garriga M.
Gómez-Ramiro M.
Soler V.
Vázquez M.
Gomes S.P.
Hospital Clínic , Psychiatry Barcelona, Spain
* Corresponding author.
Keywords: serotoninergic toxicity, sertraline, E-POSTER PRESENTATION: INTELLECTUAL DISABILITY

Conflict of interest:

No

Article ID: EPP0623
Subjects: Abstract, Mental health care - Part II

Satisfaction with services provided for people affected by severe mental illness and their family members in azerbaijan.

Ismayilova J.
National Mental Health Center of the Ministry of Health, Consulting and Treatment , Baku , Azerbaijan
Keywords: schizophrenia, services, family, satisfaction

Conflict of interest:

No

Article ID: EPP0635
Subjects: Abstract, Mental Health Care

Patients with cardiometabolic disorders associated with antipsychotics who receive pharmacological treatment for those adverse effects. Differences according to the kind of mental health unit

Olivas O. 1 *
Arnaiz A. 2
Erkoreka L. 3
Lázaro F. 4
Moreno T. 5
Varela N. 6
Basterreche N. 2
Zumarraga M. 7
Arrue A. 7
1 Osakidetza, Red De Salud Mental De Bizkaia, Gernika. Bizkaia, Spain
2 Bizkaia Mental Health Network, Zamudio Hospital, Zamudio , Spain
3 Galdakao-Usansolo Hospital, Psychiatry, Galdakao, Spain
4 Red de Salud Mental de Bizkaia, Bermeo Hospital, Bermeo. Bizkaia , Spain
5 Galdakao_Usansolo Hospital, Osakidetza, Psiquiatria, Galdakao, Spain
6 Red de Salud Mental de Bizkaia, Uipo Tratamiento Asertivo Comunitario Uribe , Getxo. Bizkaia , Spain
7 Bizkaia Mental Health Network, Neurochemical Research Unit , Barakaldo , Spain
* Corresponding author.
Keywords: antipsychotic, cardiometabolic disturbances, metabolic syndrome

Conflict of interest:

No

Article ID: EPP0637
Subjects: Abstract, Mental health care - Part III

Joint planning of discharge people with severe mental illnesses as a future model of continuity care and more quality mental health services

Racetovic G. 1 *
Latinovic M. 2
Branka S. 3
Mesic E. 4
1 Health Center Prijedor, Community Mental Health Center, Prijedor , Bosnia and Herzegovina
2 Ministry of Health and Social Welfare of Republic of Srpska, Department for Hospital Care, Banja Luka , Bosnia and Herzegovina
3 Ministry of Health and Social Welfare of Republic of Srpska, Department for Social, Family and Child Welfare, Banja Luka , Bosnia and Herzegovina
4 Asocijacija XY, Project Of Mental Health In Bosnia and Herzegovina, Sarajevo, Bosnia and Herzegovina
* Corresponding author.
Keywords: Discharge, Community, Collaboration, Hospital

Conflict of interest:

No

Article ID: EPP0657
Subjects: Abstract, Mental Health Policies

Integrating mental health in primary health care, success story (mental health gap action program (mhgap), egypt /2019)

Noby S.
General Secretariat of Mental Health & Addiction Treatment, Ministry of Health, Egypt, Director Of Mental Health Gap Action Program (mhgap) Egypt, Executive Director Of Research Unit At Gsmhat., Cairo , Egypt

Conflict of interest:

No

Article ID: EPP0684
Subjects: Abstract, Neuroscience in psychiatry - Part I

The clinical case of a 53-year-old-woman with atypical cognitive symptoms.

De Oliveira Azevedo F. 1 *
Fernandes M. 2
Barreto H. 1
Sequeira A.S. 1
1 Centro Hospitalar Lisboa Ocidental , Psiquiatria E Saúde Mental, Lisboa , Portugal
2 Centro Hospitalar Lisboa Ocidental , Neurologia, Lisboa , Portugal
* Corresponding author.
Keywords: neurology, Alzheimer, Pseudodementia, dementia

Conflict of interest:

No

Article ID: EPP0685

Dementia or pseudodementia in a 45-year-old woman with a zolpidem dependence

De Oliveira Azevedo F. 1 *
Fernandes M. 2
Spínola C. 1
Almeida C. 1
1 Centro Hospitalar de Lisboa Ocidental , Departamento De Psiquiatria E Saúde Mental, Lisboa , Portugal
2 Centro Hospitalar Lisboa Ocidental , Neurologia, Lisboa , Portugal
* Corresponding author.
Keywords: neurology, frontotemporal dementia, Pseudodementia, Zolpidem

Conflict of interest:

No

Article ID: EPP0688

‘Research domain criteria’ in context: pi3k/pdk1/pkb signaling pathway, fear, anxiety and cognitive deficits

Giménez-Llort L. 1 *
Santana-Santana M. 2
Bayascas J.R. 3
1 Universitat Autònoma de Barcelona , Institute Of Neuroscience & Department of Psychiatry and Forensic Medicine, Barcelona , Spain
2 Universitat Autònoma de Barcelona , Institute Of Neuroscience & Dept Of Psychiatry and Forensic Medicine, Barcelona , Spain
3 Universitat Autònoma de Barcelona , Institut Of Neurosciences & Department of Biochemistry and Molecular Biology, Barcelona , Spain
* Corresponding author.
Keywords: Animal models, Negative Valence Systems, Behavioral neuroscience, RDoC

Conflict of interest:

No

Article ID: EPP0706
Subjects: Abstract, Obsessive-Compulsive Disorder

Cognitive-behavioural conceptualization of e-poster presentation: obsessive-compulsive disorder

Fernandes Santos C. *
Miranda C.
Medeiros A.B.
Hospital Garcia de Orta, Department of Psychiatry and Mental Health, Almada , Portugal
* Corresponding author.
Keywords: obsessive-compulsive disorder, cognitive-behavioural model, cognitive-behavioural therapy

Conflict of interest:

No

Article ID: EPP0713
Subjects: Abstract, Old age psychiatry - Part I

Systematic review of clinical evidence for the use of antipsychotic medication in the treatment of delirium

Facucho-Oliveira J. *
Espada-Santos P.
Esteves-Sousa D.
Albuquerque M.
Costa M.
Moutinho A.
Cintra P.
Mendonça L.
Tropa J.
Hospital de Cascais, Department of Psychiatry, Cascais , Portugal
* Corresponding author.
Keywords: Delirium, antipsychotic medication

Conflict of interest:

No

Article ID: EPP0716

How are we using twitter when referring to alzheimer?

Alvarez-Mon M. 1 *
Llavero-Valero M. 2
Pereira-Sanchez V. 3
Sanchez-Bayona R. 1
Vallejo-Valdivielso M. 4
Monserrat J. 5
Lahera G. 6
Asunsolo Del Barco A. 5
Alvarez-Mon M. 5
1 University of Navarra Clinic , Department of Psychiatry, Pamplona , Spain
2 University of Navarra Clinic , Department of Endocrinology, Madrid , Spain
3 New York University , Langone Child Study Center, New York , United States of America
4 University of Navarra Clinic, Child & Adolescent Psychiatry Unit , Department of Psychiatry and Clinical Psychology, Pamplona , Spain
5 University of Alcala , Department of Medicine and Medical Specialties, Madrid , Spain
6 University of Alcala , School Of Medicine, Alcalá de Henares , Spain
* Corresponding author.
Keywords: Twitter, Advocacy, Prevention, Alzheimer

Conflict of interest:

No

Article ID: EPP0746
Subjects: Abstract, Old age psychiatry - Part IV

Description of the long-term individual course of neuropsychiatric symptoms in people with alzheimer’s and lewy body dementia

Vik-Mo A. 1 *
Borda M. 2
Aarsland D. 3
1 Centre of Age-related Medicine, Centre Of Age-related Medicine, Randaberg , Norway
2 King's College London , Department of Old Age Psychiatry, Institute Of Psychiatry, Psychology and Neuroscience, United Kingdom
3 Centre of Age-related Medicine, Stavanger University Hospital , Norway, stavanger , Norway
* Corresponding author.
Keywords: dementia, affective, psychosis, Neuropsychiatric

Conflict of interest:

No

Article ID: EPP0750

Brain aging and frontal lobe disorders - a review of the current evidence

Valido R. *
Ferreira P.
Hospital de Magalhães Lemos , Psychiatry, Porto , Portugal
* Corresponding author.
Keywords: brain aging, executive impairment, frontotemporal regions, Prefrontal Cortex

Conflict of interest:

No

Article ID: EPP0753
Subjects: Abstract, Oncology and Psychiatry

Depression and cancer - a bidirectional relationship

Barranha R. *
Quarenta J.
Pinto H.
Centro Hospitalar do Tâmega e Sousa , Department of Psychiatry and Mental Health, Penafiel , Portugal
* Corresponding author.
Keywords: major depressive disorder, cancer, Survival, Inflammation

Conflict of interest:

No

Article ID: EPP0770
Subjects: Abstract, Others - Part I

Mach-iv (test of machiavellianism): psychometric properties and normative data from the portuguese population

Esteves-Pereira M. 1 *
Azeredo A. 2
Moreira D. 3
Almeida F. 4
Brandão I. 5
1 University of Porto , Faculty Of Medicine, Porto , Portugal
2 University of Porto , Laboratory Of Neuropsychophysiology- Faculty Of Psychology and Educational Sciences, Porto , Portugal
3 University of Porto , Laboratory Of Neuropsychophysiology- Faculty Of Psychology and Educational Sciences, Porto , Porto, Portugal
4 Maia University Institute , Social and Behavioral Sciences Department, Maia, Portugal
5 University of Porto Faculdade De Medicina, Porto , Portugal
* Corresponding author.
Keywords: MACH-IV, Test of Machiavellianism, Portuguese population, Personality Tests

Conflict of interest:

No

Article ID: EPP0773

Psychiatric conditions in sports: when stigma wins the match

Caetano F.
Hospital de Magalhães Lemos , Serviço De Internamento B, Porto , Portugal
Keywords: Stigma, Sports psychiatry

Conflict of interest:

No

Article ID: EPP0794
Subjects: Abstract, Others - Part III

Heterogeneity in the diagnostics of attention deficit hyperactivity disorder diagnosis in the Czech Republic among adults

Vňuková M.
Ptáček R. *
Raboch J.
First Faculty of Medicine, Psychiatry, Prague , Czech Republic
* Corresponding author.
Keywords: ADHD, diagnosis, structured assessment

Article ID: EPP0802

Mental health and climate change in the literature

Ramos S. *
Sousa D.
Mendes J.
Unidade Local de Saúde da Guarda , E.P.E., Psychiatry , Guarda , Portugal
* Corresponding author.
Keywords: Climate Change, mental health

Conflict of interest:

No

Article ID: EPP0805
Subjects: Abstract, Pain

Fibromyalgia and bipolar affective disorder: a significant influence?

Brás J. *
Sousa R.
Costa A.
Guedes B.
Costa A.
Gil N.
Centro Hospitalar Tondela-Viseu , Department of Psychiatry and Mental Health, VISEU , Portugal
* Corresponding author.
Keywords: Bipolar disorder, fibromyalgia

Conflict of interest:

No

Article ID: EPP0819
Subjects: Abstract, Personality and personality disorders - Part II

Personality traits among regular and occasional pokémon go users

Giménez Palomo A. 1 *
Anmella G. 2
Pinzon Espinosa J. 3
Gomes S.P. 4
Pacchiarotti I. 5
1 Hospital Clínic , Psychiatry, Barcelona , Spain
2 Hospital Clinic, University of Barcelona, IDIBAPS, CIBERSAM , Schizophrenia Unit , Institute Of Neuroscience , Barcelona , Spain
3 Hospital Clinic de Barcelona, Institut Clinic De Neurociencias , Barcelona , Spain
4 Hospital Clinic de Barcelona , Psychiatry, Barcelona , Spain
5 Bipolar and Depressive Disorders Unit, Hospital Clínic de Barcelona , Psychiatry, Barcelona , Spain
* Corresponding author.
Keywords: Pokémon Go, Personality traits, substance use, sociability

Conflict of interest:

No

Article ID: EPP0822

Relationship between dispositional optimism and road rage in adult

Syed A. 1
Asif A. 2
Khalifa J. 3 *
Arif M. 4
Butt S. 5
1 Bahria University , Karachi Campus , Institute Of Professional Psychology, North Nazimabad Town , Karachi , Pakistan
2 Bahria University , Karachi Campus, Institute Of Professional Psychology, Nazimabad Block , Karachi, Pakistan
3 Bahria University , Karachi Campus , Institute Of Professional Psychology, DHA Phase , Karachi , Pakistan
4 Bahria University , Karachi Campus , Institute Of Professional Psychology, Malir Cantt, Gulshan-e-Umair , Karachi , Pakistan
5 Bahria University Karachi Campus , Institute Of Professional Psychology, Karachi , Pakistan , Pakistan
* Corresponding author.
Keywords: Optimistic Personality, Adult, Road rage, Aggressive Driving

Conflict of interest:

No

Article ID: EPP0841
Subjects: Abstract, Posttraumatic Stress Disorder

Complex post-traumatic stress disorder in combatants in the view of the eleven edition of the international classification of diseases

Haydabrus A.
V.N.Karazin Kharkiv National University , Clinical Neurology, Psychiatry and Narcology., Kharkiv , Ukraine
Keywords: complex post-traumatic stress disorder, post-traumatic stress disorder, Adaptation, combatants

Conflict of interest:

No

Article ID: EPP0875
Subjects: Abstract, Promotion of Mental Health

Mental health promotion strategies for migrants and refugees in Europe: main outcomes, recommendations and lessons learnt from myhealth european project

Evangelidou S. 1 *
Schouler-Ocak M. 2
Gionakis N. 3
Movsisyan N. 4
Qureshi A. 1
Collazos F. 1
1 Vall d’Hebron University Hospital / Research Institute , Psychiatry, Barcelona , Spain
2 Charité – Universitätsmedizin Berlin, Psychiatrische Universitätsklinik Der Charité , Berlin , Germany
3 Syn Eirmos NGO of Social Solidarity Astiki Etairia E, Scientific Coordination , Athens , Greece
4 St. Anne’s University Hospital Brno , Cardiology, Brno, Czech Republic
* Corresponding author.
Keywords: migrants and refugees and Europe and mental health promotion

Conflict of interest:

No

Article ID: EPP0888
Subjects: Abstract, Psychoneuroimmunology

Enhanced bacterial translocation in major depressive disorder patients.

Alvarez-Mon M. 1 *
Gomez A. 2
Orozco A. 3
Lahera G. 4
Sosa M.D. 2
Diaz D. 2
Auba E. 1
Albillos A. 2
Monserrat J. 2
Alvarez-Mon M. 2
1 University of Navarra Clinic , Department of Psychiatry, Pamplona , Spain
2 University of Alcala , Department of Medicine and Medical Specialties, Madrid , Spain
3 Hospital Principe de Asturias , Division Of Psychiatry, Madrid , Spain
4 University of Alcala , School Of Medicine, Alcalá de Henares , Spain
* Corresponding author.
Keywords: bacterial translocation, leaky gut, major depressive disorder, gut-brain axis

Conflict of interest:

No

Article ID: EPP0890

Autoimmunity makes me crazy: psychiatric manifestations of autoimmune encephalitis.

Espada-Santos P. *
Facucho-Oliveira J.
Albuquerque M.
Costa M.
Esteves-Sousa D.
Mendonça L.
Cintra P.
Tropa J.
Moutinho A.
Hospital de Cascais Dr. José de Almeida, Departamento De Psiquiatria E Saúde Mental, Alcabideche , Portugal
* Corresponding author.
Keywords: Autoimmune encephalitis, autoantibodies, neuropsychiatric symptoms

Conflict of interest:

No

Article ID: EPP0913
Subjects: Abstract, Psychopharmacology and pharmacoeconomics - Part I

Lithium poisoning: a silent killer?

Caetano F. 1 *
Samouco A. 2
Araújo M. 3
Priscila C. 1
Frias P. 4
Barros M. 4
Carvalho S. 1
1 Hospital de Magalhães Lemos , Serviço De Internamento B, Porto , Portugal
2 Unidade Local de Saúde do Norte Alentejano , Department of Psychiatry and Mental Health, Portalegre , Portugal
3 Hospital de Magalhães Lemos , Serviço De Internamento C, Porto , Portugal
4 Hospital de Magalhães Lemos , Psychiatry, Porto , Portugal
* Corresponding author.
Keywords: Toxicity, SILENT, Bipolar, lithium

Conflict of interest:

No

Article ID: EPP0916

A chart-based association study of newer antidepressant medication use and weight change in a population in Lebanon

El-Khoury J. 1 *
Beayno A. 1
Noufi P. 1
Bejjani M. 2
Abed Al Ahad M. 3
Majari G. 4
Ahmad A. 1
1 American University of Beirut Medical Center , Psychiatry, Beirut , Lebanon
2 American University of Beirut Medical Center, Clinical Research Institute research Institute , Beirut , Lebanon
3 American University of Beirut Medical Center, Clinical Research Institute , Beirut , Lebanon
4 American University of Beirut Medical Center , Radiology, Beirut , Lebanon
* Corresponding author.
Keywords: Lebanon, Antidepressants, Weight, pharmacology

Article ID: EPP0929
Subjects: Abstract, Psychopharmacology and pharmacoeconomics - Part II

Applicability of pharmacogenomic testing in the treatment of major depressive disorder

Ramos S. *
Sousa D.
Mendes J.
Unidade Local de Saúde da Guarda, E.P.E., Psychiatry, Guarda, Portugal
* Corresponding author.
Keywords: major depressive disorder, Pharmacogenomics

Conflict of interest:

No

Article ID: EPP0936

A second generation of neuroleptic malignant syndrome - a case report

Vieira Mendes C. *
Ferreira P.
ULSAM , Psiquiatria E Saúde Mental, viana do castelo, Portugal
* Corresponding author.
Keywords: antipsychotics, malignant neuroleptic syndrome, second generation

Conflict of interest:

No

Article ID: EPP0948
Subjects: Abstract; Psychosurgery & stimulation methods (ECT, TMS, VNS, DBS)

Neuroplastic effects of electroconvulsive therapy may be mediated by reduced IL-6 levels

Belge J.-B. 1 *
Van Diermen L. 1
Sienaert P. 2
Vansteelandt K. 2
Parizel P. 1
Sabbe B. 3
Constant E. 4
De Timary P. 4
Morrens M. 1
Coppens V. 1
Schrijvers D. 3
Vaneijndhoven P. 5
1 Antwerp University , Adult Psychiatry, Antwerp , Belgium
2 Katholieke Universiteit Leuven , Adult Psychiatry, Leuven , Belgium
3 Antwerp University , Adult Psychiatry, antwerp , Belgium
4 Université Catholique de Louvain , Adult Psychiatry, Brussels , Belgium
5 Radboud University , Adult Psychiatry, Nijmegen , Netherlands
* Corresponding author.
Keywords: Major Depression Disorder, Interleukin 6, Electroconvulsive Therapy, Hippocampus

Conflict of interest:

No

Article ID: EPP0989
Subjects: Abstract, Rehabilitation and psychoeducation - Part I

Physical exercise interventions in first-episode psychosis

Gasparinho R. *
Fernandes N.
Santos N.
Martins M.
Alho A.
Ferreira L.
Ribeirinho A.
Hospital Distrital de Santarém , Psychiatry and Mental Health, Santarém , Portugal
* Corresponding author.
Keywords: physical exercise, first-episode psychosis, Non-pharmacological interventions, rehabilitation

Conflict of interest:

No

Article ID: EPP1018
Subjects: Abstract, Schizophrenia and other psychotic disorders - Part I

When psychosis faces twitter.

Alvarez-Mon M. 1 *
Llavero-Valero M. 2
Sanchez-Bayona R. 1
Pereira-Sanchez V. 3
Vallejo-Valdivielso M. 4
Monserrat J. 5
Lahera G. 6
Asunsolo Del Barco A. 5
Alvarez-Mon M. 5
1 University of Navarra Clinic , Department of Psychiatry, Pamplona , Spain
2 University of Navarra Clinic , Department of Endocrinology, Madrid , Spain
3 New York University , Langone Child Study Center, New York , United States of America
4 University of Navarra Clinic , Child & Adolescent Psychiatry Unit, Department of Psychiatry and Clinical Psychology, Pamplona , Spain
5 University of Alcala , Department of Medicine and Medical Specialties, Madrid , Spain
6 University of Alcala , School Of Medicine, Alcalá de Henares , Spain
* Corresponding author.
Keywords: schizoprenia, psychosis, Twitter, Stigma

Conflict of interest:

No

Article ID: EPP1052
Subjects: Abstract, Schizophrenia and other psychotic disorders - Part IV

High doses of aripiprazol once-monthly in the treatment of patients with severe schizophrenia. A 36-month follow-up

Fernandez-Miranda J.J. 1 *
Diaz-Fernandez S. 2
1 AGC SM-V-HUCAB-SESPASTURIAN MENTAL HEALTH SERVICE , Centro De Tratamiento Integral, GIJON , Spain
2 AGC-S MENTAL-V-SESPAASTURIAN MENTAL HEALTH SERVICE , Centro De Tratamiento Integral, GIJON , Spain
* Corresponding author.
Keywords: schizophrenia, antipsychotics, treatment outcomes, doses

Conflict of interest:

No

Article ID: EPP1056

Clinical features of late-onset schizophrenia: a case report

Fernandes Santos C. *
Miranda C.
Medeiros A.B.
Hospital Garcia de Orta , Department of Psychiatry and Mental Health, Almada , Portugal
* Corresponding author.
Keywords: schizophrenia, psychosis, late-onset

Conflict of interest:

No

Article ID: EPP1063
Subjects: Abstract, Schizophrenia and other psychotic disorders - Part IX

Functioning in schizophrenia: a worldwide delphi study

Nuño L. 1 *
Barrios M. 2
Rojo E. 3
Gómez-Benito J. 2
Guilera G. 2
1 Hospital Clínic de Barcelona , Addictions Unit. Psychiatry and Psychology Service, Barcelona , Spain
2 University of Barcelona , Department of Social Psychology and Quantitative Psychology, Barcelona , Spain
3 Hospital Benito Menni , Casm , Sisters Hospitallers , Sant Boi de Llobregat , Spain
* Corresponding author.
Keywords: functioning, schizophrenia, Delphi study, WHO regions

Conflict of interest:

No

Article ID: EPP1064

Functioning in schizophrenia from the perspective of occupational therapists: an international delphi study

Nuño L. 1 *
Barrios M. 2
Rojo E. 3
Gómez-Benito J. 2
Guilera G. 2
1 Hospital Clínic de Barcelona , Addictions Unit. Psychiatry and Psychology Service, Barcelona , Spain
2 University of Barcelona , Department of Social Psychology and Quantitative Psychology, Barcelona , Spain
3 Hospital Benito Menni , Casm , Sisters Hospitallers , Sant Boi de Llobregat , Spain
* Corresponding author.
Keywords: Occupational therapy, schizophrenia, functioning, Delphi study

Conflict of interest:

No

Article ID: EPP1083
Subjects: Abstract, Schizophrenia and other psychotic disorders - Part V

Let the games begin

Gonçalves Cerejeira J. *
Santos Carrasco I.D.L.M.
Capella C.
Rodríguez E.
Queipo De Llano M.
Gonzaga A.
Guerra G.
De Lorenzo M.
Gómez M.
De Uribe N.
Santiago O.
Hospital Clinic of Valladolid , Psychiatry, Valladolid , Spain
* Corresponding author.
Keywords: Periventricular heterotopia, first-psychotic episode

Conflict of interest:

No

Article ID: EPP1085
Subjects: Abstract, Schizophrenia and other psychotic disorders - Part VI

The study of needs and burden associated with severe mental illness in azerbaijan.

Ismayilova J.
National Mental Health Center of the Ministry of Health , Consulting and Treatment, Baku , Azerbaijan
Keywords: schizophrenia, Burden, Severe mental illness, needs

Conflict of interest:

No

Article ID: EPP1103
Subjects: Abstract, Schizophrenia and other psychotic disorders - Part VII

Impact of a first psychosis episode program on functional variables after two years of follow-up

Matilde M. 1 *
García De Jalón E. 2
Otero M. 3
Pêreda N. 4
Ariz M.C. 5
1 Servicio Navarro de Salud, Salud Mental Primeros Episodios , Pamplona , Spain
2 Complejo Hospitalario de Navarra , Servicio De Psiquiatría, Pamplona , Spain
3 OSASUMBIDEA, Salud Mental Primeros Episodios , Pamplona , Spain
4 osasunbidea, Salud Mental Primeros Episodios , Pamplona , Spain
5 Servicio Navarro de Salud, Servicio De Psiquiatría. Primeros Episodios ., Pamplona , Spain
* Corresponding author.

Conflict of interest:

No

Article ID: EPP1106
Subjects: Abstract, Schizophrenia and other psychotic disorders - Part VIII

Acylcarnitines and amino acids change with schizophrenia

Mednova I. 1 *
Kassakin M. 2
Chernonosov A. 2
Kornetova E. 3
Semke A. 3
Bokhan N. 4
Koval V. 2
Ivanova S. 1
1 Mental Health Research Institute, Tomsk National Research Medical Center of the Russian Academy of Sciences , Laboratory Of Molecular Genetics and Biochemistry, Tomsk , Russian Federation
2 Institute of Chemical Biology and Fundamental Medicine, Siberian Branch of the Russian Academy of Sciences, Core Facility Of Mass Spectrometric Analysis , Novosibirsk , Russian Federation
3 Mental Health Research Institute, Tomsk National Research Medical Center, Russian Academy of Sciences , Department of Endogenous Disorders, Tomsk , Russian Federation
4 Mental Health Research Institute, Tomsk National Research Medical Center of the Russian Academy of Sciences, Addictive States Department , Tomsk , Russian Federation
* Corresponding author.
Keywords: Acylcarnitine, Amino acid, schizophrenia, Metabolomics

Article ID: EPP1107

“Every breath you take…I’ll be watching you” - celebrities’ stalking and its connection with mental illness

Melo-Ribeiro P. 1 *
Santos F. 2
Batista S. 3
Mota-Oliveira M. 3
1 Centro Hospitalar Universitário do Algarve , Departamento De Psiquiatria E Saúde Mental De Faro, Faro , Portugal
2 Centro Hospitalar Universitário do Algarve , Psychiatry, Faro , Portugal
3 Centro Hospitalar Universitário do Algarve , Departamento De Psiquiatria E Saúde Mental - Faro, Faro , Portugal
* Corresponding author.
Keywords: stalking, Personality disorders, mental illness, Delusional disorder

Conflict of interest:

No

Article ID: EPP1109

The role of the gut microbiome in schizophrenia: current evidence

Miguel A. *
Basto M.
Mendes E.
Centro Hospitalar Vila Nova de Gaia/Espinho , Serviço De Psiquiatria, Vila Nova de Gaia , Portugal
* Corresponding author.
Keywords: schizophrenia, Gut, Microbiome, psychosis

Conflict of interest:

No

Article ID: EPP1115

Falling in love with whom? A case report and brief literature review of coexistence of de clerambault and fregoli syndromes.

Moura N. 1 *
Santana L. 2
Facucho J. 3
Domingues S. 4
Esteves-Sousa D. 5
Canas-Simião H. 2
1 Centro Hospitalar Lisboa Ocidental , Psychiatry, Lisboa , Portugal
2 Centro Hospitalar Lisboa Ocidental , Departamento De Psiquiatria E Saúde Mental, Lisbon , Portugal
3 Hospital de Cascais Dr. José de Almeida , Psychiatry, Alcabideche , Portugal
4 Centro Hospitalar Médio Tejo , Psychiatry, Tomar , Portugal
5 Hospital de Cascais , Department of Psychiatry, Cascais , Portugal
* Corresponding author.

Conflict of interest:

No

Article ID: EPP1116

SocialMIND® improves attentional performance in schizophrenia spectrum disorder (SDD) patients.

Muñoz-Sanjose A. 1 *
Mediavilla R. 2
Pinto García A. 3
Vidal-Villegas M.P. 4
Louzao Í.I. 5
Aguirre P. 5
Román Mazuecos E.M. 2
Sánchez P. 6
Pastor Haro J. 7
Rodríguez-Vega B. 2
1 La Paz University Hospital Institute for Health Research (IdiPAZ) , Psychiatry , Clinical Psychology and Mental Health , Madrid , Spain
2 La Paz University Hospital Research Health Institute (IdiPAZ) , Psychiatry , Clinical Psychology and Mental Health , Madrid , Spain
3 Fundación para la Investigación Biomédica del Hospital Universitario La Paz, Mental Health , Madrid , Spain
4 La Paz University Hospital Biomedical Research Foundation (F.I.B.H.U.L.P.) , Psychiatry , Clinical Psychology and Mental Health, MADRID , Spain
5 Hospital Universitario La Paz , Psychiatry , Clinical Psychology and Mental Health , Madrid , Spain
6 La Paz University Hospital , Psychiatry , Clinical Psychology and Mental Health , Madrid , Spain
7 Hospital Universitario La Paz, Psiquiatría Y Salud Mental , Madrid , Spain
* Corresponding author.
Keywords: Schizophrenia Spectrum Disorder, attention, CPT-IP, Mindfulness

Conflict of interest:

No

Article ID: EPP1121
Subjects: Abstract, Schizophrenia and other psychotic disorders - Part X

Common genes in neurodevelopment and immune-inflammatory pathways impacted in schizophrenia?

Ramoz N. 1 *
Gorwood P. 2
1 INSERM , Institute Of Psychiatry and Neuroscience Of Paris , Paris , France
2 Sainte-Anne Hospital , Cmme Department, Paris , France
* Corresponding author.
Keywords: copy number variations, genetic correlation, genome-wide association study, microRNA

Conflict of interest:

No

Article ID: EPP1126

Diagnostic stability in the first-episode psychosis

Santos Silva L. 1 *
Duarte M. 1
Araújo R. 1
Barbosa M. 1
Miranda J. 1
Almeida C. 2
1 Centro Hospitalar de Leiria, Serviço Psiquiatria E Saúde Mental , Leiria , Portugal
2 Centro Hospitalar de Leiria , Psychiatry, Leiria , Portugal
* Corresponding author.
Keywords: First-Episode, psychosis, diagnosis

Conflict of interest:

No

Article ID: EPP1142
Subjects: Abstract, Schizophrenia and other psychotic disorders - Part XII

Severe catatonia following quetiapine abrupt withdrawal

Valido R. *
Frias P.
Caldas F.
Ferreira P.
Hospital de Magalhães Lemos, Psychiatry, Porto, Portugal
* Corresponding author.
Keywords: Catatonia, Withdrawal catatonia, Psychotic Depression, Quetiapine

Conflict of interest:

No

Article ID: EPP1155
Subjects: Abstract, Sexual Medicine and Mental Health

Managing antidepressant-associated sexual dysfunction

Esteves-Sousa D. 1 *
Moura N. 2
Albuquerque M. 1
Facucho-Oliveira J. 3
Espada-Santos P. 1
Mendonça L. 1
Tropa J. 1
1 Hospital de Cascais Dr. José de Almeida , Departamento De Psiquiatria E Saúde Mental, Alcabideche , Portugal
2 Centro Hospitalar Lisboa Ocidental , Department of Psychiatry, Lisboa , Portugal
3 Hospital de Cascais , Department of Psychiatry, Cascais , Portugal
* Corresponding author.
Keywords: sexual dysfunction, Antidepressants, Depressive disorder, side-effects

Conflict of interest:

No

Article ID: EPP1200
Subjects: Abstract, Suicidology and suicide prevention - Part III

Oedipism – from sophocles’s tragedy to present days

Gasparinho R. *
Fernandes N.
Martins M.
Santos N.
Alho A.
Ferreira L.
Ribeirinho A.
Hospital Distrital de Santarém, Psychiatry and Mental Health, Santarém, Portugal
* Corresponding author.
Keywords: self-injurious behavior, Oedipism, Eye Enucleation, Self-enucleation

Conflict of interest:

No

Article ID: EPP1210

Autolytic ideation among medical residents in spain: a cross-sectional study

Hernández-Calle D. 1 *
Cano Arenas A. 1
Carracedo Sanchidrián D. 1
Bravo-Ortiz M.F. 2
1 Hospital Universitario La Paz , Psychiatry, Madrid , Spain
2 La Paz University Hospital Research Health Institute (IdiPAZ) , Psychiatry , Clinical Psychology and Mental Health , Madrid , Spain
* Corresponding author.
Keywords: Suicidal ideation, Medical Residents

Conflict of interest:

No

Article ID: EPP1263
Subjects: Abstract; Women, gender and mental health - Part II

Women, loneliness, false stigmas and management on frequent attenders of primary care system

Giménez-Llort L. 1 *
Vidal-Llaudes M. 2
Odriozola-García A. 2
Nunes F. 2
1 Universitat Autònoma de Barcelona , Institute Of Neuroscience & Department of Psychiatry and Forensic Medicine, Barcelona , Spain
2 Universitat Autònoma de Barcelona , Department of Psychiatry and Forensic Medicine, Barcelona , Spain
* Corresponding author.
Keywords: women, Gender Medicine, Loneliness, primary care

Conflict of interest:

No

Article ID: EPP1270

The role of perfectionism and repetitive negative thinking in prenatal OC phenomena

Mota D. 1 *
Pereira A.T. 2
Xavier S. 2
Azevedo J. 3
Soares M.J. 2
Marques C. 3
Marques M. 4
Araújo A. 3
Macedo A. 2
1 Centro Hospitalar Universitario de Coimbra , Psiquiatria, Coimbra , Portugal
2 University of Coimbra , Faculty Of Medicine, Department of Psychological Medicine, Coimbra , Portugal
3 Faculty of Medicine, University of Coimbra , Institute Of Psychological Medicine, Coimbra , Portugal
4 Coimbra Hospital and University Centre , Clinical Psychology, Coimbra , Portugal
* Corresponding author.
Keywords: prenatal, obsessive-compulsive disorder, perfectionism

Conflict of interest:

No

Article ID: EPP1293
Subjects: Intellectual Disability

Features of complex support for children with a delay in the development of psychogenic genesis in an inclusive practice

Bratkova M.
Afanasyeva Y. *
Sidneva Y.
Russian Federation
* Corresponding author.
Keywords: children with a delay in mental development (DMD); inclusive education, developmental delay of psychogenic origin

Article ID: EPV0015
Subjects: Abstract, E-Poster Viewing Addictive disorders

Valproate to the rescue: treating a psychotic episode with polymorphic features

Facão R. 1 *
Corvacho M. 2
Borges L. 1
Canelas P. 1
Silva J. 1
1 Centro Hospitalar Universitário do Algarve , Psiquiatria, Portimão , Portugal
2 Centro Hospitalar Universitário do Algarve , Departamento De Psiquiatria E Saúde Mental, Faro , Portugal
* Corresponding author.
Keywords: psychosis, Valproate

Conflict of interest:

No

Article ID: EPV0034

Gamma-hydroxybutyric acid (GHB) detoxification: a review.

Rodríguez E.
Hospital Clínic Barcelona , Institute of Neuroscience, Barcelona , Spain
Keywords: Gamma-hydroxybutyric acid, Dependence, withdrawal, Detoxification

Conflict of interest:

No

Article ID: EPV0035

Psychopharmacological management of the abstinence and craving stages of alcohol dependence: evidence-based recommendations

Sousa Martins P. *
Bettencourt M.
Unidade Local de Saúde do Nordeste , E.P.E., Departamento De Psiquiatria E Saúde Mental, Bragança , Portugal
* Corresponding author.
Keywords: Alcohol, abstinence, craving, psychopharmacology

Conflict of interest:

No

Article ID: EPV0043

Alcoholic hallucinosis, a case report.

Dhiver Cantalejo Y. 1 *
Sánchez Amador G. 2
Padilla Romero P. 2
1 Hospital General Nuestra Señora del Prado , Psychiatry , Talavera de la Reina , Toledo , Spain
2 Hospital General Nuestra Señora del Prado , Psychiatry, Talavera, Toledo , Spain
* Corresponding author.
Keywords: Alcoholic hallucinosis, delirium tremens, schizophrenia, Dipsomaniac drinker

Conflict of interest:

No

Article ID: EPV0044

Amphetamine psychosis, a case report

Dhiver Cantalejo Y. 1 *
Padilla Romero P. 2
Sánchez Amador G. 2
Mancha Heredero E. 3
Pérez Castellano M.T. 2
1 Hospital General Nuestra Señora del Prado , Psychiatry , Talavera de la Reina , Toledo , Spain
2 Hospital General NuestraSeñora del Prado , Psychiatry, Talavera, Toledo , Spain
3 Hospital ClínicoUniversitario , Psiquiatria, Valladolid , Spain
* Corresponding author.
Keywords: ADH, psychosis, Amphetamine, psychostimulants

Conflict of interest:

No

Article ID: EPV0045

Psychopharmacologic automedication in medical residents in spain: a cross-sectional study.

Hernández-Calle D. 1 *
Carracedo Sanchidrián D. 1
Cano Arenas A. 1
Bravo-Ortiz M.F. 2
1 Hospital Universitario La Paz , Psychiatry, Madrid , Spain
2 La Paz University Hospital Research Health Institute (IdiPAZ) , Psychiatry , Clinical Psychology And Mental Health , Madrid , Spain
* Corresponding author.
Keywords: Medical Residents, Automedication

Conflict of interest:

No

Article ID: EPV0090
Subjects: Abstract, Anxiety Disorders and Somatoform Disorders

Takotsubo myocardiophaty and anxiety: a case report

Carpio Garcia L. *
Martín Villarroel C.
Dominguez Cutanda J.
Belmonte Garcia G.
Fernandez Torija Daza M.
HOSPITAL PROVINCIAL DE LA MISERICORDIA , PsiquiatrÍa (consultas Externas), TOLEDO , Spain
* Corresponding author.
Keywords: Anxiety, stress, Tako-Tsubo cardiomyopathy

Conflict of interest:

No

Article ID: EPV0091

Resistant anxiety and affective instability as a marker of abuses in the past: a case report

Carpio Garcia L. *
Martín Villarroel C.
Dominguez Cutanda J.
Belmonte Garcia G.
Fernandez Torija Daza M.
HOSPITAL PROVINCIAL DE LA MISERICORDIA , PsiquiatrÍa (consultas Externas), TOLEDO , Spain
* Corresponding author.
Keywords: Affective instability, trauma, Anxiety, Abuse

Conflict of interest:

No

Article ID: EPV0093

Specific phobia, a case report

Dhiver Cantalejo Y. 1 *
Sánchez Amador G. 2
Padilla Romero P. 2
1 Hospital General Nuestra Señora del Prado , Psychiatry , Talavera de la Reina , Toledo , Spain
2 Hospital General Nuestra Señora del Prado , Psychiatry, Talavera, Toledo , Spain
* Corresponding author.
Keywords: cognitive behavioral, phobia, Anxiety, SSRI

Conflict of interest:

No

Article ID: EPV0101

Psychological factors predict electrodiagnostic studies results

Papadopoulou M. 1
Michopoulos I. 2 *
Tsivgoulis G. 3
Palaiodimou L. 3
Bregianni M. 3
Voumvourakis K. 3
1 University of West Attica , Department of Physiotherapy, Athens , Greece
2 National and Kapodistrian University of Athens , 2nd Department of Psychiatry, Attikon General Hospital , Athens , Greece
3 National and Kapodistrian University of Athens , 2nd Department of Neurology, Attikon Hospital , Athens , Greece
* Corresponding author.
Keywords: Electrodiagnostic studies, Psychological factors, HADS, WHODAS

Conflict of interest:

No

Article ID: EPV0112

Revisiting psychogenic non-epileptic seizures: a literature review

Santos Silva L. *
Almeida C.
Barbosa M.
Miranda J.
Duarte M.
Centro Hospitalar de Leiria, Serviço Psiquiatria E Saúde Mental , Leiria , Portugal
* Corresponding author.
Keywords: psychogenic seizures, non-epileptic seizures, seizures

Conflict of interest:

No

Article ID: EPV0116

Psychosomatic symptoms and affective disorders: an exploratory data analysis.

Fernández R. 1 *
López N. 2
Mesa N. 1
Caballero Martínez L. 1
1 HM CINAC, Servicio De Psiquiatría Y Psicología Clínica Hm Hospitales , Móstoles , Spain
2 Universidad San Pablo CEU , Facultad De Medicina, Móstoles , Spain
* Corresponding author.
Keywords: anxiety disorders, depressive disorders, somatoform disorders, exploratory data analysis

Conflict of interest:

No

Article ID: EPV0121
Subjects: Abstract, Bipolar Disorders

Steroid-induced mania. Clinical features and treatment. About a case.

Carrajo Garcia C. *
Rodriguez De Lorenzo M.
Alonso Sánchez E.
Loeck De Lapuerta C.
HOSPITAL UNIVERSITARIO RAMON Y CAJAL , Psychiatry , MADRID , Spain
* Corresponding author.
Keywords: olanzapine, corticosteroids, mania, oncological patient

Conflict of interest:

No

Article ID: EPV0135

Is functional impairment stable in patients with bipolar disorder and schizophrenia over time?

López-Villarreal A. 1 *
Jiménez-López E. 2
Sánchez-Morla E.M. 3
Aparicio A.I. 4
Rodriguez-Jimenez R. 3
Vidal Pérez P. 5
Santos J.L. 4
1 Hospital Virgen de La Luz , Departament Psychiatry, Cuenca , Spain
2 Universidad de Castilla La Mancha, Health And Social Research Center Of Cuenca , Cuenca , Spain
3 Hospital Universitario 12 de Octubre, Madrid, Spain, Department of Psychiatry, Madrid, Spain
4 Hospital Virgen de la Luz , Department of Psychiatry, Cuenca , Spain
5 Consorci Corporació Sanitària Parc Taulí de Sabadell , Psychiatry, Barcelona , Spain
* Corresponding author.
Keywords: Bipolar disorder, follow up, psychosocial functioning, schizophrenia

Conflict of interest:

No

Article ID: EPV0146

Impulsivity in bipolar disorder. Comorbidity with attention deficit hyperactivity disorder.

Rodríguez-Quiroga A. 1 *
Nava P. 2
Matas Ochoa A.M. 1
Martinez De Velasco R. 2
Banzo C. 2
Mora F. 2
1 Hospital Universitario Infanta Leonor , Psychiatry, Madrid , Spain
2 Hospital Infanta Leonor , Psychiatry, Madrid , Spain
* Corresponding author.
Keywords: Bipolar disorder, Tics, ADHD, Impulsivity

Conflict of interest:

No

Article ID: EPV0152
Subjects: Abstract, Bipolar Disorders

Blood test to diagnose bipolar disorder using rna editing-related biomarkers

Chimienti F. *
Salvetat N.
Dupré P.
Dubuc B.
Cayzac C.
Checa-Robles F.
Patel V.
Weissmann D.
ALCEDIAG/SYS2Diag , Montpellier , France
* Corresponding author.
Keywords: diagnostic, RNA editing, Bipolar disorder

Conflict of interest:

No

Article ID: EPV0188
Subjects: Abstract, Child and Adolescent Psychiatry

Emotional regulation in infants born after a threatening preterm labour: a prospective study.

Campos-Berga L. 1 *
Moreno-Giménez A. 2
Sahuquillo-Leal R. 2
García-Blanco A. 3
1 UNIVERSITY AND POLYTECHNIC HOSPITAL LA FE, VALENCIA, Psychiatry And Psychology, VALENCIA , Spain
2 University of Valencia , Psychology , VALENCIA , Spain
3 University of Valencia// Health Research Institute La Fe , Psychology// Neonatology, VALENCIA , Spain
* Corresponding author.
Keywords: threatening preterm labor (TPL), prenatal stress, neurodevelopment, emotional regulation

Conflict of interest:

No

Article ID: EPV0191

The boy who needed more than pills

Carrajo Garcia C. 1 *
Loeck De Lapuerta C. 1
Rodriguez De Lorenzo M. 1
Alonso Sánchez E. 1
Del Sol Calderón P. 2
1 HOSPITAL UNIVERSITARIO RAMON Y CAJAL , Psychiatry , MADRID , Spain
2 HOSPITAL UNIVERSITARIO PUERTA DE HIERRO MAJADAHONDA , Psychiatry , MADRID , Spain
* Corresponding author.
Keywords: psychotherapy, Adoption, Asperger, attention deficit

Conflict of interest:

No

Article ID: EPV0198

Mental health care needs and access to specialized services in children with mental disorders

Díaz-Castro L. 1 *
Márquez-Caraveo M. 2
Cabello-Rangel H. 3
1 National Institute of Psychiatry Ramon de la Fuente Muñiz , Direction Of Epidemiological And Psychosocial Research, Mexico City , Mexico
2 Child Psychiatric Hospital “Dr. Juan N. Navarro” ., Division Of Research, Mexico City , Mexico
3 Psychiatric Hospital “Fray Bernardino Álvarez”., Division Of Diagnostic Assistants, Mexico City , Mexico
* Corresponding author.
Keywords: Mental health needs, Access, Children, mental disorders

Conflict of interest:

No

Article ID: EPV0199

Diagnostic stability in early-onset first-episode psychosis

Fernandez E. Dutilh *
Maga;a M. Mendez
Hospital Virgen del Puerto , Unidad De Hospitalizacion Breve, Paraje de Valcorchero, Spain
* Corresponding author.
Keywords: Schizoaffective disorder, first-episode psychosis, psychosis, early-onset psychosis

Conflict of interest:

No

Article ID: EPV0202

Relation between trauma and psychosis at transition period (late adolescente and young adult)

Garcia E. 1 *
Barredo P. 2
1 Sescam, Psychiatry, Toledo , Spain
2 Sescam, Psychology, Toledo , Spain
* Corresponding author.
Keywords: trauma, psychosis, Transition, Multiintervention

Conflict of interest:

No

Article ID: EPV0222

Generalized problematic internet use, use of social networks, and appearance schemas in late adolescence

Maia B. 1 *
Moreira H. 2
Pereira A.T. 3
Macedo A. 4
1 Universidade Católica Portuguesa , Centre For Philosophical And Humanistic Studies, Braga , Portugal
2 University of Coimbra , Center for Research In Neuropsyhology And Cognitive Behavioral Intervention, Coimbra , Portugal
3 Faculty of Medicine, University of Coimbra, Institute of Psychological Medicine , Coimbra , Portugal
4 University of Coimbra , Faculty of Medicine, Department of Psychological Medicine, Coimbra , Portugal
* Corresponding author.
Keywords: Problematic Internet Use, appearance schemas, use of social networks, Late Adolescence

Conflict of interest:

No

Article ID: EPV0223

Associations between social anxiety and avoidance, attachment styles and parental marital status, in late adolescence

Maia B. 1 *
Coelho C. 2
Marques M. 3
Carvalho F. 4
1 Universidade Católica Portuguesa , Centre For Philosophical And Humanistic Studies, Braga , Portugal
2 The Catholic University of Portugal , Faculty of Philosophy And Social Sciences, Braga , Portugal
3 Coimbra Hospital and University Centre , Clinical Psychology, Coimbra , Portugal
4 Espaço Psicológico – Consultório de Psicologia, Consultório De Psicologia, Coimbra , Portugal
* Corresponding author.
Keywords: parental marital status, attachment, Late Adolescence, social anxiety and avoidance

Conflict of interest:

No

Article ID: EPV0226

The influence of new technologies in adolescents and young people

Molina Cambra R. *
García-Poggio Fernández-Renau M.
Muñoz Domenjó A.
Ramos García E.
Martínez Fernández Á.
Bianchi Ramos F.L.
Sagarra Arruego R.
Ortega Moreno M.
Hernández Barrera M.
Hospital Universitario de Móstoles , Psiquiatría, Móstoles, Madrid , Spain
* Corresponding author.
Keywords: adolescents, young people, new technologies, behavioral addictions

Conflict of interest:

No

Article ID: EPV0238

Common aspects of behavioral disorders and related diagnoses.

Quero-Palomino V. *
Diaz-Quero I.
Muñoz-Martínez V.
Mata-Saenz B.
Gomez-Peinado A.
Torralba-Viorreta R.
Hospital General Universitario de Ciudad Real , Psychiatry, Ciudad Real , Spain
* Corresponding author.
Keywords: family intervention, pharmacotherapy, adolescents, Behavioral disorders

Conflict of interest:

No

Article ID: EPV0242

Avoidant restrictive food intake disorder: an illustrative case example

Taillefer Aguanell V. 1 *
Barquero Paz F. 2
Más Villaseñor A. 3
Plaza Yuste M. 4
Muñoz Domenjó A. 5
1 Hospital Universitario Guadalajara , Psiquiatría, Guadalajara , Spain
2 Hospital Universitario Badajoz , Psiquiatría, Badajoz , Spain
3 Hospital Nuestra señora de Sonsoles Ávila , Psiquiatría, Ávila , Spain
4 Hospital Central de la Defensa Gómez Ulla , Psychiatry And Mental Health Department, Madrid , Spain
5 Hospital universitario móstoles , Psiquiatría, Madrid , Spain
* Corresponding author.
Keywords: ARFID, Feeding, disorder, childhood

Conflict of interest:

No

Article ID: EPV0251

Is there a specific developmental organization of graphomotor gesture in school aged children with handwriting disabilities?

Lopez C. 1
Brunel H. 2
Vaivre-Douret L. 3 *
1 Faculty of Medicine, University of Paris , Paris Descartes ; INSERM Unit1178/1018-CESP, Necker-Enfants Malades University Hospital, AP-HP, France , Department of Child Psychiatry, Paris, France, Paris, France
2 INSERM Unit1178/1018-CESP, Necker-Enfants Malades University Hospital , AP-HP ; Department of Engineering of Automatic Systems and Industrial Computing, Polytecnic University of Ca, Department of Child Psychiatry, Paris, France, Paris , France
3 Faculty of Medicine, University of Paris , Paris Descartes ; INSERM Unit1178/1018-CESP, Necker-Enfants Malades University Hospital, AP-HP, France ; France University Institute (IUF), Department of Child Psychiatry, Paris, France, Paris, France
* Corresponding author.
Keywords: handwriting gesture, child, development, handwriting disabilities

Conflict of interest:

No

Article ID: EPV0266

The mental health of minors residing in centers

Gómez González C.L. 1 *
Imma I.P. 2
Marta C.F. 2
1 Hospital Sant Joan de Déu of Barcelona , Child And Adolescent Psychiatry And Psychology Department, Esplugues de Llobregat, Barcelona , Spain
2 Sant Joan de Deu Hospital , Child/youth Mental Health Centre, Barcelona , Spain
* Corresponding author.
Keywords: fostercare, behavioral problems, residential centers, adolescents

Conflict of interest:

No

Article ID: EPV0282

Some gifted children are diagnosed with developmental coordination disorder (DCD) but have they identical clinical features compared to typical children with DCD?

Vaivre-Douret L. 1 *
Hamdioui S. 2
Cannafarina A. 2
1 Faculty of Medicine, University of Paris , Paris Descartes; INSERM UMR 1178/1018-CESP, and University Institute (IUF), Department of Child Psychiatry, Necker–enfants-malades University Hospital, Ap-hp, Paris, France
2 Faculty of Medicine, University of Paris , Paris Descartes, INSERM Unit 1178/1018-CESP, Department of Child Psychiatry, Necker–enfants-malades University Hospital, Ap-hp, Paris, France
* Corresponding author.
Keywords: high intellectual quotient, IQ, gifted children, developmental coordination disorder

Conflict of interest:

No

Article ID: EPV0284
Subjects: Abstract, Classification of Mental Disorders

The schizo-obsessive spectrum. About a clinical case.

Rodríguez-Quiroga A. 1 *
Nava P. 2
Matas Ochoa A.M. 1
Martínez De Velasco R. 1
Banzo C. 2
Mora F. 2
1 Hospital Universitario Infanta Leonor , Psychiatry, Madrid , Spain
2 Hospital Infanta Leonor , Psychiatry, Madrid , Spain
* Corresponding author.
Keywords: schizophrenia, magical thinking, obsessive-compulsive disorder, clozapine

Conflict of interest:

No

Article ID: EPV0285

The IQ profile (homogeneous versus heterogeneous) in children with high intellectual potential (HIP): what are the links with some emotional, interactional and neurodevelopmental disorders?

Vaivre-Douret L. 1 *
Planche P. 2
1 Faculty of Medicine, University of Paris , Paris Descartes ; INSERM UMR 1178/1018-CESP, Necker–Enfants-Malades University Hospital, AP-HP, France University Institute (IUF), Department of Child Psychiatry, Paris , France
2 Faculty of letters and Human Sciences, Brest University , Laboratory Cread (ea 3875), Brest , France
* Corresponding author.
Keywords: High intellectual potential child, homogeneous IQ profile, heterogeneous IQ profile, Neurodevelopmental disorders

Conflict of interest:

No

Article ID: EPV0287

Dimensional analysis of adolescent attention-deficit/hyperactivity disorder

Fernández-Martín P. 1 *
León J.J. 1
Rodríguez-Herrera R. 1
Cánovas R. 2
Martínez De Salazar A. 1 3
Cobos-Sánchez L. 1 4
Sánchez-Santed F. 1 2
Flores P. 1 2
1 University of Almería & Health Research Centre , Department of Psychology, Faculty of Psychology, Almería , Spain
2 InPaula, Institute of Child Neurorehabilitation , Department of Neuropsychology, Almería , Spain
3 Torrecárdenas University Hospital, Child And Adolescent Mental Health Unit, Almería , Spain
4 Sol de Portocarrero High School, Department of Educational Guidance, Almería , Spain
* Corresponding author.
Keywords: Dimensional diagnosis, attention, ADHD, inhibitory control

Conflict of interest:

No

Article ID: EPV0289

A critical overview of the schizophrenia-obsessive-compulsive disorder interphase

Ibáñez Vizoso J.E. *
Rodado León B.
Saiz González M.D.
Hospital Universitario Clínico San Carlos, Instituto De Psiquiatría Y Salud Mental , Madrid , Spain
* Corresponding author.
Keywords: obsessive-compulsive symptoms, schizophrenia, schizoobsesive, Obsessive-Compulsive Disorder

Conflict of interest:

No

Article ID: EPV0305
Subjects: Abstract, Comorbidity / Dual Pathologies

Ticking time-bomb

Loeck De Lapuerta C. 1 *
Rodriguez De Lorenzo M. 2
Carrajo Garcia C. 3
Alonso Sánchez E. 4
1 RAMON Y CAJAL UNIVERSITY HOSPITAL, Psychiatry , MADRID , Spain
2 Hospital Universitario Ramon y Cajal, Psychiatry, Madrid , Spain
3 HOSPITAL UNIVERSITARIO RAMON Y CAJAL , Psychiatry , MADRID , Spain
4 HOSPITAL UNIVERSITARIO RAMON Y CAJAL , Psychiatry, Madrid , Spain
* Corresponding author.
Keywords: brugada, schizophrenia, dual patology, antipshycotic drugs

Conflict of interest:

No

Article ID: EPV0313

Stabilization of a patient with a major depressive sindrome with psychotic and obsessive symptoms and hiperthyroidism

Vilas Boas J. *
Torres S.
Centro Hospitalar Barreiro Montijo, Psychiatry And Mental Health , Barreiro , Portugal
* Corresponding author.
Keywords: Depressive, Obessive, Psychotic, Hiperthyroidism

Conflict of interest:

No

Article ID: EPV0338

Is there a role for aripiprazole in alcohol use disorders?

Gasparinho R. 1 *
Nogueira V. 2
Alho A. 1
Santos N. 1
Martins M. 1
Fernandes N. 1
Ferreira L. 1
Teixeira J. 3
1 Hospital Distrital de Santarém, Psychiatry And Mental Health, Santarém , Portugal
2 Centro Hospitalar Psiquiátrico de Lisboa, Psiquiatria, Lisboa , Portugal
3 Centro Hospitalar Psiquiátrico de Lisboa, Alcoologia E Novas Dependências (and), Lisboa , Portugal
* Corresponding author.
Keywords: aripiprazole, alcoholism, Alcohol Use Disorders

Conflict of interest:

No

Article ID: EPV0355
Subjects: Abstract, Consultation Liaison Psychiatry and Psychosomatics

Cannabinoid hyperemesis in liaison psychiatry. Case report.

Rodriguez De Lorenzo M. 1 *
Alonso Sánchez E. 1
Carrajo Garcia C. 2
Loeck De Lapuerta C. 3
Correa A. 4
Guillama Henriquez A. 1
Del Sol Calderón P. 5
1 Hospital Universitario Ramon y Cajal , Psychiatry, Madrid , Spain
2 HOSPITAL UNIVERSITARIO RAMON Y CAJAL , Psychiatry , MADRID , Spain
3 RAMON Y CAJAL UNIVERSITY HOSPITAL, Psychiatry , MADRID , Spain
4 Ramon y Cajal Hospital, Psychiatry, Madrid , Spain
5 HOSPITAL UNIVERSITARIO PUERTA DE HIERRO MAJADAHONDA, Psychiatry , MADRID , Spain
* Corresponding author.
Keywords: cannabis, Liaison Psychiatry, shower, cannabinoid hyperemesis

Conflict of interest:

No

Article ID: EPV0364

Korsakoff síndrome. Clinical features and treatment. Case report

Carrajo Garcia C. 1 *
Rodriguez De Lorenzo M. 1
Alonso Sánchez E. 2
Loeck De Lapuerta C. 1
Del Sol Calderón P. 3
1 HOSPITAL UNIVERSITARIO RAMON Y CAJAL, Psychiatry , MADRID , Spain
2 HOSPITAL UNIVERSITARIO RAMON Y CAJAL, Psychiatry, Madrid , Spain
3 HOSPITAL UNIVERSITARIO PUERTA DE HIERRO MAJADAHONDA, Psychiatry , MADRID , Spain
* Corresponding author.
Keywords: Alcohol, Cognitive evaluation, thiamine, amnesic-confabulatory syndrome

Conflict of interest:

No

Article ID: EPV0365

Tacrolimus associated with reversible posterior encephalopathy in liver transplant recipient

Carrajo Garcia C. 1 *
Alonso Sánchez E. 2
Loeck De Lapuerta C. 1
Rodriguez De Lorenzo M. 1
Del Sol Calderón P. 3
1 HOSPITAL UNIVERSITARIO RAMON Y CAJAL, Psychiatry , MADRID , Spain
2 HOSPITAL UNIVERSITARIO RAMON Y CAJAL, Psychiatry, Madrid , Spain
3 HOSPITAL UNIVERSITARIO PUERTA DE HIERRO MAJADAHONDA, Psychiatry , MADRID , Spain
* Corresponding author.
Keywords: solid organ transplant, calcineurin inhibitors, confabulations, tempospatial disorientation

Conflict of interest:

No

Article ID: EPV0368

Psychotic symptoms in parkinson disease. About a case

Rodríguez-Quiroga A. 1 *
Nava P. 2
Matas Ochoa A.M. 1
Martinez De Velasco R. 2
Banzo C. 2
Mora F. 2
1 Hospital Universitario Infanta Leonor, Psychiatry, Madrid , Spain
2 Hospital Infanta Leonor, Psychiatry, Madrid , Spain
* Corresponding author.
Keywords: Parkinson disease, psychosis, visual hallucinations, dopamine agonists

Conflict of interest:

No

Article ID: EPV0369

Cardiac rehabilitation. A program that saves lives

Rodríguez-Quiroga A. 1 *
Nava P. 2
Matas Ochoa A.M. 1
Martinez De Velasco R. 2
Banzo C. 2
Villacañas M. 1
Mora F. 2
1 Hospital Universitario Infanta Leonor, Psychiatry, Madrid , Spain
2 Hospital Infanta Leonor, Psychiatry, Madrid , Spain
* Corresponding author.
Keywords: Dépression, ischemic heart disease, adjustment disorder, acute myocardial infarction

Conflict of interest:

No

Article ID: EPV0372

Sexual improvement in hepatitis C infected patients cured after direct acting antivirals: one-year follow-up.

Cavero M. 1 *
Navines R. 1
Rodriguez-Tajes S. 2
Lens S. 2
Bartres C. 2
Nácar L. 1
Pla A. 2
Miralpeix A. 2
Gonzalez M. 3
Sarquella J. 3
Sastre L. 2
Martin-Santos R. 1
Forns X. 2
Mariño Z. 2
1 Hospital Clinic, UB, IDIBAPS, CIBERSAM, Psychiatry And Psychology, Barcelona , Spain
2 Hospital Clinic, UB, IDIBAPS, CIBERehd , Liver Unit, Barcelona , Spain
3 Fundació Puigvert, Andrology Department, Barcelona , Spain
* Corresponding author.
Keywords: antivirals, Sexual improvement, hepatitis C

Conflict of interest:

No

Article ID: EPV0375

Mutism culminating in a diagnosis of creutzfeldt-jakob disease - a case report

Machado E. *
Fonseca L.
Fonseca J.
Rodrigues R.
Simões S.
Hospital da Senhora da Oliveira, Psychiatry And Mental Health, Guimarães , Portugal
* Corresponding author.
Keywords: mutism, creutzfeldtjakobdisease

Conflict of interest:

No

Article ID: EPV0378

Complications of alcohol use disorder: a view from the liaison psychiatry service

Muñoz-Calero P. 1 *
Muñoz Domenjó A. 2
Rodríguez Criado N. 3
Ramos García E. 2
1 Hospital Universitario de Móstoles, Psychiatry , MADRID , Spain
2 Hospital Universitario de Móstoles, Psiquiatría, Móstoles, Madrid , Spain
3 Hospital Infantil Universitario Niño Jesús , Child And Adolescent Psychiatry And Psychology Department, Madrid , Spain
* Corresponding author.
Keywords: complications, Alcohol, Liaison Psychiatry

Conflict of interest:

No

Article ID: EPV0392
Subjects: Abstract, Cultural Psychiatry

Culture and personality disorders-a case series.

Cuevas I. *
Fontecha Banegas L.
Moreno Alonso I.
Blanco Prieto M.
Hospital Universitario Principe de Asturias , Psiquiatria, Alcala de Henares , Spain
* Corresponding author.
Keywords: Personality disorders, cross-cultural

Conflict of interest:

No

Article ID: EPV0412
Subjects: Abstract, Depressive Disorders

¿Dead or alive? cotard syndrome: a case report.

Algarín Moriana M.P. 1 *
Guzmán Del Castillo A.H. 1
López Sánchez E.J. 2
Sánchez Gayango A. 2
Velarde Pedraza G. 1
1 Hospital Universitario Nuestra Señora Virgen de Valme , Psiquiatría, Sevilla , Spain
2 Hospital Nuestra Señora de Valme, Psiquiatría, Sevilla , Spain
* Corresponding author.
Keywords: cotard, syndrome, electroconvulsive, therapy

Conflict of interest:

No

Article ID: EPV0452

Depression and academic learning in children with high intellectual potential (HIP): is there a relationship?

Vaivre-Douret L.
Faculty of Medicine, University of Paris , Paris Descartes ; INSERM UMR 1178/1018-CESP, Necker–Enfants-Malades University Hospital, AP-HP, France University Institute (IUF), Department of Child Psychiatry, Paris , France
Keywords: Dépression, Academic learning, High intellectual potential child, IQ

Conflict of interest:

No

Article ID: EPV0489
Subjects: Abstract, Eating Disorders

Neuropsychological aspects in eating disorders: the rey complex figure test

Mata-Saenz B. 1
Muñoz-Martinez V. 1
Diaz-Quero I. 1 *
Quero-Palomino V. 1
Belda-Moreno G. 1
Leon-Velasco M. 1
Beato-Fernandez L. 1
Rodriguez-Cano T. 2
1 Hospital General Universitario de Ciudad Real , Psychiatry, Ciudad Real , Spain
2 Servicios Centrales SESCAM, Coordinación Regional De Salud Mental, Toledo , Spain
* Corresponding author.
Keywords: Neuropsychology, eating disorders, The Rey Complex Figure Test, Executive Function

Conflict of interest:

No

Article ID: EPV0490

Assessment of autistic traits in adolescents with eating disorders.

Muñoz-Martínez V.
Mata-Saenz B.
Rodriguez-Cano T.
Diaz-Quero I. *
Quero-Palomino V.
Beato-Fernandez L.
Hospital General Universitario de Ciudad Real , Psychiatry, Ciudad Real , Spain
* Corresponding author.
Keywords: Autism, Eating Disorders, Adolescents, Outcome, Treatment.

Conflict of interest:

No

Article ID: EPV0506

How dissociation influences in eating disorders prognosis

Muñoz-Martínez V.
Mata-Saenz B.
Rodriguez-Cano T.
Quero-Palomino V. *
Diaz-Quero I.
Hospital General Universitario de Ciudad Real , Psychiatry, Ciudad Real , Spain
* Corresponding author.
Keywords: Eating Disorders, Dissociative Experiences. Attitude Towards Change.

Conflict of interest:

No

Article ID: EPV0507

Neuropsychological impairment associated with depression in a sample of patients with eating disorders

Mata-Saenz B. 1
Muñoz-Martinez V. 1
Quero-Palomino V. 1 *
Diaz-Quero I. 1
Leon-Velasco M. 1
Belda-Moreno G. 1
Rodriguez-Cano T. 2
Beato-Fernandez L. 1
1 Hospital General Universitario de Ciudad Real , Psychiatry, Ciudad Real , Spain
2 Servicios Centrales SESCAM, Coordinación Regional De Salud Mental, Toledo , Spain
* Corresponding author.
Keywords: Dépression, Neuropsychology, Cognitive impairment, Eating Disorders

Conflict of interest:

No

Article ID: EPV0511

Is family therapy really helping our patients with eating disorders? A 6-year retrospective study

Martins Ramos B. *
Coutinho F.
Delgado A.
Barbosa D.
Osório E.
Brandão I.
São João Hospital and University Center , Psychiatry And Mental Health Department, Porto , Portugal
* Corresponding author.
Keywords: eating disorder, family therapy, Anorexia nervosa, Bulimia nervosa

Conflict of interest:

No

Article ID: EPV0547
Subjects: Abstract, Emergency Psychiatry

Corticosteroid-induced mania in the emergency room: a case report.

Bermejo Pastor A. 1 *
Galerón Guzmán R. 1
Herrero Pellón E. 1
Huete Naval M. 1
Albarracín Marcos P. 1
Alberdi Páramo Í. 2
1 Hospital Clínico San Carlos., Institute of Psychiatry And Mental Health., Madrid , Spain
2 Hospital Universitario de la Princesa , Psychiatry Department, Madrid , Spain
* Corresponding author.
Keywords: mania, corticosteroids, Bipolar, Emergency

Conflict of interest:

No

Article ID: EPV0549

The lunatic association between full moon and psychiatric emergency care visits

Hernández-Calle D. 1 *
Garcia Cardenal A. 1
Kollias G. 1
Suárez Lorenzo A. 1
Bravo-Ortiz M.F. 2
1 Hospital Universitario La Paz , Psychiatry, Madrid , Spain
2 La Paz University Hospital Research Health Institute (IdiPAZ) , Psychiatry, Clinical Psychology And Mental Health, Madrid , Spain
* Corresponding author.

Conflict of interest:

No

Article ID: EPV0566

Flumazenil response in hepatic encephalopathy: a case report.

Fernández R. 1 *
Harkous C. 1
Blanco R. 2
Rodríguez A. 3
Vizcaíno Da Silva M. 3
Mendez Gonzalez O. 2
1 HM CINAC, Servicio De Psiquiatría Y Psicología Clínica Hm Hospitales, Móstoles , Spain
2 HOSPITAL UNIVERSITARIO PUERTA DE HIERRO MAJADAHONDA , Psychiatry, MADRID , Spain
3 Centro de Salud San Carlos, Psiquiatría, San Lorenzo del Escorial , Spain
* Corresponding author.
Keywords: flumazenil, hepatic encephalopathy, benzodiacepines, GABA

Conflict of interest:

No

Article ID: EPV0577
Subjects: Abstract, Epidemiology and Social Psychiatry

Back pain treatment for patients with mental disorders

Lachina M. 1 *
Ljungdalh P. 2
Garvik O. 1
Stenager E. 3
Schiøttz-Christensen B. 4
Nørgård B. 1
1 Center for Clinical Epidemiology, Odense University Hospital and University of Southern Denmark , Department of Clinical Epidemiology, Odense C , Denmark
2 The Spine Centre of Southern Denmark , Medical Research Department, Middelfart , Denmark
3 Psychiatric Research Unit, Department of Regional Health Research, University Of Southern Denmark , Åbenrå , Denmark
4 The Spine Centre of Southern Denmark and Institute of Regional Health Science , Medical Research Department, Middelfart , Denmark
* Corresponding author.
Keywords: back pain, pre-existing mental disorders

Conflict of interest:

No

Article ID: EPV0583

Cyberbullies or cybervictims: does gender and age matter?

Sahli L. 1 *
Bourgou S. 1
Askri W. 1
Haj Amor S. 2
Fakhfakh R. 2
Belhadj A. 1
1 Mongi Slim Hospital , Child Psychiatry Department, Tunis , Tunisie, Tunisia
2 Medical Center, Birsa, Tunis , Tunisia, 3 National Public Health Institute , Preventive Medecine Department, Ariana , Tunisia
* Corresponding author.
Keywords: adolescent, aggression

Conflict of interest:

No

Article ID: EPV0589

Academic burnout in third year students of medicine.

Quero-Palomino V. *
Torralba-Viorreta R.
Gomez-Peinado A.
Mata-Saenz B.
Muñoz-Martínez V.
Diaz-Quero I.
Hospital General Universitario de Ciudad Real , Psychiatry, Ciudad Real , Spain
* Corresponding author.
Keywords: academic burnout, academic stressors, Medicine students, mental health

Conflict of interest:

No

Article ID: EPV0590

Academic stress and coping strategies in medical university students

Quero-Palomino V. *
Gomez-Peinado A.
Torralba-Viorreta R.
Mata-Saenz B.
Muñoz-Martínez V.
Diaz-Quero I.
Hospital General Universitario de Ciudad Real , Psychiatry, Ciudad Real , Spain
* Corresponding author.
Keywords: coping style, medical university students, academic stress, coping strategies

Conflict of interest:

No

Article ID: EPV0612
Subjects: Abstract, Ethics and Psychiatry

Advance decision to refuse treatment in anorexia nervosa. A case report.

Moreno Alonso I. 1 *
García Guixot C. 1
Fontecha Banegas L. 1
Cuevas I. 2
1 Hospital Universitario Principe de Asturias , Psiquiatria, Alcala de Henares , Spain
2 Hospital Universitario Principe de Asturias , Psiquiatria, Alcalá de Henares , Spain
* Corresponding author.
Keywords: ‘TCA’, ‘ethical’, ‘advance decision’

Conflict of interest:

No

Article ID: EPV0620

Restraint in psychiatry: the perception of caregivers

Messedi N. *
Charfeddine F.
Aribi L.
Amami O.
Hédi Chaker university Hospital , Department of Psychiatry (b), Sfax , Tunisia
* Corresponding author.
Keywords: restraint, feelings of caregivers, Psychiatry, patients’ impact

Conflict of interest:

No

Article ID: EPV0621

Restraint in psychiatry: assessing the knowledge of nurses and trainee doctors

Messedi N. *
Chamseddine A.
Aribi L.
Charfeddine F.
Amami O.
Hédi Chaker university Hospital , Department of Psychiatry (b), Sfax , Tunisia
* Corresponding author.
Keywords: Physical restraint, nurses and trainee doctors’ knowledge, practice towards physical restraint

Conflict of interest:

No

Article ID: EPV0629
Subjects: Abstract, Forensic Psychiatry

Suspicion of chemical submission in a hospital emergency department: a case report

Rentero Martin D. 1 *
Parra Gonzalez A. 1
Zamora Banegas B. 2
Santana Florido V. 2
Eguía Barbarin J.L. 1
1 Hospital 12 de Octubre, Psiquiatría, Madrid , Spain
2 Hospital 12 de Octubre, Psiquiatría, madrid , Spain
* Corresponding author.
Keywords: Drugs, forensic, Chemical submission, Toxicology

Conflict of interest:

No

Article ID: EPV0665
Subjects: Abstract, Intellectual Disability

Depression in patients with mental retardation: a case report

Dehanov S. *
Ferreira T.
Hospital Prof. Doutor Fernando Fonseca , Psychiatry Department, Amadora , Portugal
* Corresponding author.
Keywords: mental retardation, Dépression

Conflict of interest:

No

Article ID: EPV0689
Subjects: Abstract, Mental Health Care

Evaluation of a coordination program between mental health and primary care

Diaz-Quero I. *
Mata-Saenz B.
Muñoz-Martínez V.
Quero-Palomino V.
Beato-Fernandez L.
Hospital General Universitario de Ciudad Real , Psychiatry, Ciudad Real , Spain
* Corresponding author.
Keywords: mental health care, epidemiology and social psychiatry, Quality Management

Conflict of interest:

No

Article ID: EPV0700

Stigmatizing attitudes of mental health professionals and their familiarity with mental disease.

Del Olmo F. 1 *
Martinez A.C. 2
Gonzalez-Blanco M. 3
Rebolleda C. 4
1 CLINICA SAN MIGUEL, HERMANAS HOSPITALARIAS , Medical Director, Madrid , Spain
2 CLINICA SAN MIGUEL, HERMANAS HOSPITALARIAS , Crps Arturo Soria, Madrid , Spain
3 CLINICA SAN MIGUEL, HERMANAS HOSPITALARIAS , MrAravaca, MADRID , Spain
4 CLINICA SAN MIGUEL, HERMANAS HOSPITALARIAS , Crps Aranjuez, ARANJUEZ , Spain
* Corresponding author.
Keywords: Stigma, attitudes, Mental Health Professionals

Conflict of interest:

No

Article ID: EPV0748
Subjects: Abstract, Neuroimaging

Is it mandatory to perform systematically a brain computed tomography in first-episode psychosis?

Nova Marqués J.A. *
Hernández Huerta D.
Alonso Cano E.B.
University Hospital Ramon y Cajal , Psychiatry Department, Madrid , Spain
* Corresponding author.
Keywords: neuroimaging, first-episode psychosis, brain computed-tomography

Conflict of interest:

No

Article ID: EPV0783
Subjects: Abstract, Neuroscience in Psychiatry

Neurofeedback to treat mild cognitive decline: what scientific literature says

Suárez-Gómez M. 1 *
Suárez-Gómez A. 2
Suárez-González F. 3
1 Unidade Local de Saúde do Baixo Alentejo , Psychiatry, Beja , Portugal
2 Centro de Saúde, Comunitary Nursery , Borba , Portugal
3 Centro de Salud San Roque , Practice Medicine, Badajoz , Spain
* Corresponding author.
Keywords: neurofeedback, Mild cognitive impairment, old age psychiatry, treatment

Conflict of interest:

No

Article ID: EPV0786
Subjects: Abstract, Obsessive-Compulsive Disorder

Neurological soft signs in obsessive-compulsive disorder

Fernandes Santos C. 1 *
Canelas Da Silva I. 2
1 Hospital Garcia de Orta , Department of Psychiatry And Mental Health, Almada , Portugal
2 Hospital Vila Franca de Xira , Department of Psychiatry, Vila Franca de Xira , Portugal
* Corresponding author.
Keywords: obsessive-compulsive disorder, neurological soft signs

Conflict of interest:

No

Article ID: EPV0787

Refractory obsessive-compulsive disorder. A case report.

Franch C.M. 1 *
Gómez Martínez R. 1
Serrano García A. 1
Calzada Miranda E. 1
Ugidos Fernandez A. 2
Alonso De La Torre A. 2
1 CAULE , Psychiatry, Leon , Spain
2 CAULE , Psychiatry, León , Spain
* Corresponding author.
Keywords: Refractory, Psychosurgery, Neurosurgery, ocd

Conflict of interest:

No

Article ID: EPV0788

Depression or obsessive compulsive disorder suicidal subtype? A case report.

Jiménez Cabañas M. *
Sánchez Amores M.
Chiva San Román L.
Huete Naval M.
Pérez Navarro V.
Carrascosa Carrascosa C.
Hospital Universitario Clínico San Carlos, Instituto De Psiquiatría Y Salud Mental , Madrid , Spain
* Corresponding author.
Keywords: obsessive-compulsive disorder, ocd, Suicidal OCD, Suicide

Conflict of interest:

No

Article ID: EPV0791

Effect of family dynamics in patients with severe obsessive-compulsive disorder.

García-González P. *
Rodríguez Muro E.
Hospital de Día de Villaverde, Psychiatry, Madrid , Spain
* Corresponding author.
Keywords: DAY HOSPITAL, obsessive-compulsive disorder, systemic therapy

Conflict of interest:

No

Article ID: EPV0798
Subjects: Abstract, Old Age Psychiatry

Is exercise effective in reducing symptoms of depression in alzheimer’s dementia? a systematic literature review

Banerjee L.V. 1 *
De Souza S. 2
1 University of Bristol , Medical School, Bristol , United Kingdom
2 Somerset Partnership NHS Foundation Trust, Old Age Psychiatry , Taunton , United Kingdom
* Corresponding author.
Keywords: dementia, Dépression, Exercise, treatment

Conflict of interest:

No

Article ID: EPV0808

Charles bonnet syndrome as the first stage of dementia development. A case presentation.

Manzanero Estopiñan R. *
Pérez Da Silva C.
University Hospital Ramon y Cajal , Psychiatry Department, Madrid , Spain
* Corresponding author.
Keywords: Charles-Bonnet syndrome, Lewy body dementia, case report, visual hallucinations

Conflict of interest:

No

Article ID: EPV0812

Use of visual hallucinations characteristics to help in the differential diagnosis between charles bonnet syndrome and lewy body dementia initial stage.

Pérez Da Silva C. 1 *
Manzanero Estopiñan R. 2
1 University Hospital Ramon y Cajal , Psychiatry Department, Madrid , Spain
2 Hospital Universitario Ramon y Cajal , Psychiatry , Madrid , Spain
* Corresponding author.
Keywords: visual hallucinations, differential diagnosis, Charles-Bonnet syndrome, Lewy body dementia

Conflict of interest:

No

Article ID: EPV0815

Communication skills with the elderly patients

Suárez-Gómez M. 1 *
Suárez-González F. 2
Suárez-Gómez A. 3
1 Unidade Local de Saúde do Baixo Alentejo , Psychiatry, Beja , Portugal
2 Centro de Salud San Roque , Practice Medicine, Badajoz , Spain
3 Centro de Saúde, Comunitary Nursery , Borba , Portugal
* Corresponding author.
Keywords: Geriatric Psychiatry, Adherence, Communication, old age

Conflict of interest:

No

Article ID: EPV0834

Neuropsychiatric symptoms in a intracranial lipome: a clinical case

Suárez-Gómez M. 1 *
Gonçalves Pereira P. 2
Rodrigues P. 1
1 Unidade Local de Saúde do Baixo Alentejo , Psychiatry, Beja , Portugal
2 Algarve Medical School , Portugal, Neuroradiology , Faro , Portugal
* Corresponding author.
Keywords: neuroimaging, lipoma, old age psychiatry, Neuropsychiatric symptoms

Conflict of interest:

No

Article ID: EPV0846
Subjects: Abstract, Oncology and Psychiatry

Interventions on distress in terminally ill cancer patients: the state of the art

Ramos S. 1 *
Tavares M.A. 2
1 Unidade Local de Saúde da Guarda , E.P.E., Psychiatry , Guarda , Portugal
2 Instituto Português de Oncologia do Porto FG , EPE, Psycho-oncology, Porto , Portugal
* Corresponding author.
Keywords: Existencial distress

Conflict of interest:

No

Article ID: EPV0861
Subjects: Abstract, Others

The relationship within the first job and the emotional intelligence in graduates of the health area of two universities in santa marta - colombia

Guardiola Esmeral A. 1
Miranda L.F. 2 *
Viloria Escobar J. 3
Pérez Correa K. 4
1 Universidad Cooperativa de Colombia, Facultad Ciencias Contables Administrativas Y De Comercio Internacional , Santa Marta , Colombia
2 Universidad del Magdalena , Facultad De Educación, Santa Marta , Colombia
3 Universidad del Magdalena , Facultad De Ciencias De La Educación, Santa Marta , Colombia
4 Universidad Cooperativa de Colombia , Facultad De Psicología, Santa Marta , Colombia
* Corresponding author.
Keywords: emotional intelligence, job, graduates, health

Conflict of interest:

No

Article ID: EPV0877

Jung and pop culture: the archetypes in harry potter.

Fontecha Banegas L.
Hospital Universitario Principe de Asturias , Psiquiatria, Alcala de Henares , Spain
Keywords: Collective Unconscious, Harry Potter, Jung, Archetypes

Conflict of interest:

No

Article ID: EPV0878

Cryothermic dermatitis artefacta in adolescence.

García Vázquez P. *
Serrano García A.
Gómez Martínez R.
Complejo Asistencial Universitario León , Psiquiatría, Leon , Spain
* Corresponding author.
Keywords: Dermatitis artefacta, Factitial dermatitis, adolescence, Impulsivity

Conflict of interest:

No

Article ID: EPV0880

Academic results as a predictor of automedication and suicidal ideation among medical residents.

Hernández-Calle D. 1 *
Carracedo Sanchidrián D. 1
Cano Arenas A. 1
Bravo-Ortiz M.F. 2
1 Hospital Universitario La Paz , Psychiatry, Madrid , Spain
2 La Paz University Hospital Research Health Institute (IdiPAZ), Psychiatry, Clinical Psychology And Mental Health , Madrid , Spain
* Corresponding author.
Keywords: Suicidal ideation, Medical Residents, Academic results, Automedication

Conflict of interest:

No

Article ID: EPV0886

Dissociation and confusion: the presentation of a susac syndrome. What the psychiatrist should know.

Pérez-Lombardo M. 1 *
Alberdi Páramo Í. 2
Bermejo Pastor A. 3
García Carpintero A. 1
Crossley López V. 1
Gimeno Álvarez M.D. 1
1 Hospital Universitario Clínico San Carlos, Instituto De Psiquiatría Y Salud Mental , Madrid , Spain
2 Hospital Clínico San Carlos., Institute of Psychiatry And Mental Health ., Madrid , Spain
3 Hospital Clínico San Carlos, Institute of Psychiatry And Mental Health , Madrid , Spain
* Corresponding author.
Keywords: diagnosis, susac, dissociation, Magnetic Resonance Imaging

Conflict of interest:

No

Article ID: EPV0891

Pathological bereavement. A case report.

Serrano N. 1 *
Infante Sanchez De Lugarnuevo M. 1
Guijarro S. 1
Díaz Fernandez M.D.C. 1
Landa E. 1
Alvites Ahumada M.D.P. 2
1 Hospital general de Tomelloso, Unidad De Salud Mental , Tomelloso , Spain
2 Complejo Hospitalario Mancha Centro, Unidad De Salud Mental Infanto Juvenil, Alcázar de San Juan (Ciudad Real) , Spain
* Corresponding author.
Keywords: bereavement, pathological bereavement, Dépression, duel

Conflict of interest:

No

Article ID: EPV0906

Preeminence of psychopathological over other medical references in names of spanish punk groups (1981-2010)

Pavez F. 1 2 *
Saura E. 3
Marset P. 4
1 University of Murcia, The International School of Doctoral Studies , Murcia , Spain
2 University of Otago Wellington, Suicide And Mental Health Research Group , Wellington , New Zealand
3 Centro de Mediación y Terapia Familiar de Águilas, Department of Psychology, Águilas , Spain
4 University of Murcia , Departamento De Ciencias Sociosanitarias, Murcia , Spain
* Corresponding author.
Keywords: Content analysis, Psychiatry in the arts, social representations, Culture and Psychiatry

Conflict of interest:

No

Article ID: EPV0918
Subjects: Abstract, Pain

Years of pain in a patient with high doses of opioid treatment: opioid induced hyperalgesia or somatic symptom disorder

Correa A. *
Ochoa Mangado E.
Caballero I.
Guillen S.
University Hospital Ramon y Cajal , Psychiatry Department, Madrid , Spain
* Corresponding author.
Keywords: hyperalgesia, opioid, somatic symptom disorder

Conflict of interest:

No

Article ID: EPV0920

Burning mouth syndrome associated to quetiapine treatment.

García Vázquez P. *
Serrano García A.
Gómez Martínez R.
Complejo Asistencial Universitario León , Psiquiatría, Leon , Spain
* Corresponding author.
Keywords: Burning mouth syndrome, Quetiapine, Xerostomia, Psychotropics

Conflict of interest:

No

Article ID: EPV0928
Subjects: Abstract, Personality and Personality Disorders

Instinct to kill: organic personality disorder or just an antisocial brain?

Borba Martins J. *
Batista S.
Trindade A.
Centro Hospitalar Universitário do Algarve , Departamento De Psiquiatria E Saúde Mental - Faro, Faro , Portugal
* Corresponding author.
Keywords: Organic personality disorder, Antisocial personality, Aggressive ideation

Conflict of interest:

No

Article ID: EPV0933

Difficulties in approaching the organic personality disorder: a case report.

Guzmán Del Castillo A.H. 1 *
Santamaría Gómez O. 1
Sánchez Gayango A. 2
Algarín Moriana M.P. 3
López Sánchez E.J. 2
Martín Talavera I. 1
1 Hospital Universitario Nuestra Señora Virgen de Valme , Psiquiatría, Sevilla , Spain
2 Hospital Nuestra Señora de Valme , Psiquiatría, Sevilla , Spain
3 Hospital Virgen de Valme, Unidad De Hospitalización De Salud Mental , Sevilla , Spain
* Corresponding author.
Keywords: Disorders, behavior, ORGANIC, Personality

Conflict of interest:

No

Article ID: EPV0934

Is neuroticism personality trait related to neurotic disorders in hiv patients?

Hernández Huerta D. 1 *
Parro Torres C. 2
Madoz Gúrpide A. 1
Ochoa Mangado E. 1
1 University Hospital Ramon y Cajal , Psychiatry Department, Madrid , Spain
2 Gregorio Marañón University Hospital, Psychiatry Institute , Madrid , Spain
* Corresponding author.
Keywords: Personality, Neuroticism, HIV, psychopathology

Conflict of interest:

No

Article ID: EPV0951

A personological dimensional perspective in pathological gambling

Nirestean A. 1 *
Lukacs E. 2
1 University of Medicine , Pharmacy, Science and Technology of Targu Mures, Psychiatry, Targu Mures , Romania
2 George Emil Palade University of Medicine , Pharmacy, Science and Technology of Targu Mures, Psychiatry, Targu Mures , Romania
* Corresponding author.
Keywords: Gambling, dimensional perspective, Personality

Conflict of interest:

No

Article ID: EPV0952

Diagnostic profile of patients treated in villaverde’s day hospital for adults

García-González P. *
Rodríguez Muro E.
Hospital de Día de Villaverde, Psychiatry, Madrid , Spain
* Corresponding author.
Keywords: personality disorder, DAY HOSPITAL, comorbidity

Conflict of interest:

No

Article ID: EPV1011
Subjects: Abstract, Psychoneuroimmunology

Pediatric acute-onset neuropyquiatric syndrome. An atypical case

Serrano N. 1 *
Díaz Fernandez M.D.C. 1
Guijarro S. 1
Infante Sanchez De Lugarnuevo M. 1
Landa E. 1
Alvites Ahumada M.D.P. 2
1 Hospital general de Tomelloso , Unidad De Salud Mental, Tomelloso , Spain
2 Complejo Hospitalario Mancha Centro , Unidad De Salud Mental Infanto Juvenil, Alcázar de San Juan (Ciudad Real), Spain
* Corresponding author.
Keywords: PANS; PANDAS; OCD,recurrent urinary infection

Conflict of interest:

No

Article ID: EPV1017
Subjects: Abstract, Psychopathology

Association between the degree of irritability caused by trigger sounds and the presence of depression and anxiety in adults with different levels of misophonia

Antonia F.T. 1 *
Giménez-Llort L. 2
1 Universidad de Barcelona , Departamento Psiquiatría, BARCELONA, Spain
2 Universidad de Barcelona , Departamento Psiquiatría, Barcelona , Spain
* Corresponding author.
Keywords: Misophonia, Anxiety, sounds, Dépression

Conflict of interest:

No

Article ID: EPV1018

Association between psychological well-being and the degree of irritability caused by trigger sounds in adults with different levels of misophonia

Antonia F.T. 1 *
Giménez-Llort L. 2
1 Universidad de Barcelona , Departamento Psiquiatría, BARCELONA , Spain
2 Universidad de Barcelona , Departamento Psiquiatría, Barcelona , Spain
* Corresponding author.
Keywords: Misophonia, trigger sounds, Adults, psychological well-being

Conflict of interest:

No

Article ID: EPV1067
Subjects: Abstract, Psychopharmacology and Pharmacoeconomics

Efficacy of aripiprazole as a corrector of risperidone-induced hyperprolactinemia.

Carrajo Garcia C. 1 *
Rodriguez De Lorenzo M. 1
Alonso Sánchez E. 2
Loeck De Lapuerta C. 1
1 HOSPITAL UNIVERSITARIO RAMON Y CAJAL , Psychiatry, MADRID, Spain
2 HOSPITAL UNIVERSITARIO RAMON Y CAJAL , Psychiatry, Madrid, Spain
* Corresponding author.

Conflict of interest:

No

Article ID: EPV1069

Does long acting antipsychotic treatment reduce antipsychotic polypharmacy at discharge from hospitalization in psychiatric inpatient units?

Pérez Da Silva C. *
Polonio Fuentes E.
Hernández Huerta D.
University Hospital Ramon y Cajal , Psychiatry Department, Madrid , Spain
* Corresponding author.
Keywords: Polypharmacy, hospitalization, long-acting injectable antipsychotics, oral antipsychotics

Conflict of interest:

No

Article ID: EPV1071

Heart rate and QT interval in patients with clozapine vs controls

Andres-Olivera P. *
Dominguez-Alvarez E.
Gamonal-Limcaoco R.S.
Bullón-Sáez A.
Garzón-De Paz M.Á.
CAUSA, Psychiatry , Salamanca , Spain
* Corresponding author.
Keywords: clozapine, QT interval, Tachycardia, heart rate

Conflict of interest:

No

Article ID: EPV1103
Subjects: Abstract; Psychosurgery & stimulation methods (ECT, TMS, VNS, DBS)

Improvement strategies in the application of electroconvulsive therapy

Martinez De Velasco R. 1 *
Nava P. 1
Rodríguez-Quiroga A. 2
Matas A. 1
Banzo C. 1
Mora F. 1
1 Hospital Infanta Leonor , Psychiatry, Madrid , Spain
2 Hospital Universitario Infanta Leonor , Psychiatry, Madrid , Spain
* Corresponding author.
Keywords: Electroconvulsive Therapy, mania, Dépression, schizophrenia

Conflict of interest:

No

Article ID: EPV1107

Electroconvulsive therapy in the treatment of comorbid obsessive-compulsive disorder and anorexia nervosa: a case report.

García Carpintero A. 1 *
Rodado León B. 1
Alberdi Páramo Í. 2
García Recio A. 1
1 Hospital Universitario Clínico San Carlos , Instituto De Psiquiatría Y Salud Mental, Madrid , Spain
2 Hospital Clínico San Carlos ., Institute of Psychiatry And Mental Health., Madrid , Spain
* Corresponding author.
Keywords: ECT, Electroconvulsive Therapy, obsessive-compulsive disorder, Anorexia nervosa

Conflict of interest:

No

Article ID: EPV1124
Subjects: Abstract, Psychotherapy

A psychoanalytical way of re-constructing the speech in psychosis

Gorgoli D. 1 *
Mitropoulos G. 2
1 Constantopouleio General Hospital of Nea Ionia , Psychiatric Department, Athens , Greece
2 Psychiatric Hospital of Attica , 5th Department, Athens , Greece
* Corresponding author.
Keywords: psychosis, lacanian, Psychoanalysis, psychoanalytical

Conflict of interest:

No

Article ID: EPV1154
Subjects: Abstract, Quality Management

Is there correlation between subjective patient satisfaction and psychiatric clinical evaluation in patients of day hospital?

García Vázquez P. *
Serrano García A.
Gómez Martínez R.
Complejo Asistencial Universitario León , Psiquiatría, Leon , Spain
* Corresponding author.
Keywords: PATIENT SATISFACTION, PSYCHIATRIC CLINICAL EVALUATION, DAY HOSPITAL

Conflict of interest:

No

Article ID: EPV1180
Subjects: Abstract, Rehabilitation and Psychoeducation

Patients perception of occupational therapy at villaverde’s day hospital.

Márquez B.
Tomás T.
García-González P. *
Hospital de Día de Villaverde, Psychiatry, Madrid , Spain
* Corresponding author.
Keywords: DAY HOSPITAL, Occupational therapy, rehabilitation

Conflict of interest:

No

Article ID: EPV1188
Subjects: Abstract, Research Methodology

Sensibility to change of mindfulness attention awareness scale (MAAS)

Pinto García A. 1 *
Muñoz-Sanjose A. 2
Vidal-Villegas M.P. 3
Mediavilla R. 4
García A. 5
Orosa-Duarte Á. 6
Palao Á. 7
Bayon C. 8
Lahera G. 9
Rodríguez-Vega B. 4
1 Fundación para la Investigación Biomédica del Hospital Universitario La Paz, Mental Health , Madrid , Spain
2 La Paz University Hospital Institute for Health Research (IdiPAZ) , Psychiatry , Clinical Psychology And Mental Health , Madrid , Spain
3 La Paz University Hospital Biomedical Research Foundation (F.I.B.H.U.L.P.) , Psychiatry , Clinical Psychology And Mental Health, MADRID , Spain
4 La Paz University Hospital Research Health Institute (IdiPAZ) , Psychiatry , Clinical Psychology And Mental Health , Madrid , Spain
5 Hospital Universitario La Paz , Psychiatry , Clinical Psychology And Mental Health , Madrid , Spain
6 La Paz University Hospital , Psychiatry, Madrid , Spain
7 HOSPITAL UNIVERSITARIO LA PAZ , Psiquiatria , MADRID , Spain
8 Autonomous University of Madrid (UAM) , School of Medicine, Madrid , Spain
9 University of Alcala , School of Medicine, Alcalá de Henares , Spain
* Corresponding author.
Keywords: MAAS, Mindfulness, Schizophrenia Spectrum Disorder

Conflict of interest:

No

Article ID: EPV1232
Subjects: Abstract, Schizophrenia and Other Psychotic Disorders

Stressful life events and the onset of psychosis

Gonçalves Cerejeira J. *
Santos Carrasco I.D.L.M.
Queipo De Llano M.
Guerra G.
Gonzaga A.
Capella C.
Rodríguez E.
De Lorenzo M.
Gómez M.
De Uribe N.
Santiago O.
Hospital Clinic of Valladolid , Psychiatry, Valladolid , Spain
* Corresponding author.
Keywords: stressful life envents, psychosis

Conflict of interest:

No

Article ID: EPV1249

Impact of a first psychosis episode program in clinical variables after two years of follow-up

Matilde M. 1 *
García De Jalón E. 2
Otero M. 3
Pêreda N. 4
Ariz M.C. 5
1 Servicio Navarro de Salud, Salud Mental Primeros Episodios , Pamplona , Spain
2 Complejo Hospitalario de Navarra , Servicio De Psiquiatría, Pamplona , Spain
3 OSASUMBIDEA, Salud Mental Primeros Episodios , Pamplona , Spain
4 osasunbidea, Salud Mental Primeros Episodios , Pamplona , Spain
5 Servicio Navarro de Salud, Servicio De Psiquiatría. Primeros Episodios ., Pamplona , Spain
* Corresponding author.

Conflict of interest:

No

Article ID: EPV1258

Symptomatology and addressability of patients with first episode psychosis at mental health services

Negrila V.I. 1 *
Culici A. 2
Negru I. 3
Moldovan R.T. 1
Rivis I.A. 4
Bredicean C. 5
1 Timișoara County Emergency Hospital , Clinic Of Psychiatry “eduard Pamfil” , Timisoara , Romania
2 Timișoara County Emergency Clinical Hospital, Clinic Of Psychiatry “eduard Pamfil” , Timișoara , Romania
3 “ Victor Babeș” University of Medicine and Pharmacy , Faculty of Medicine, Timișoara , Romania
4 High-security Psychiatric Hospital Jebel , Psychiatry , Jebel , Romania
5 “Victor Babeș” University of Medicine and Pharmacy , Neuroscience , Timișoara , Romania
* Corresponding author.
Keywords: addressability, First Episode Psychosis, hallucinations, delusions

Conflict of interest:

No

Article ID: EPV1263

Myxedema madness: psychosis may also happen. a review of symptoms through a case report.

Pérez-Lombardo M. 1 *
González Castellary A. 2
Gómez Beteta F.J. 1
Pemán Rodríguez J. 1
Ibáñez Vizoso J.E. 1
Villanueva Gallego M. 1
Jiménez Cabañas M. 1
Rodado León B. 1
Montero Hernández G. 1
Strada Herrera G. 1
1 Hospital Universitario Clínico San Carlos, Instituto De Psiquiatría Y Salud Mental , Madrid , Spain
2 Hospital Universitario La Princesa, Unidad Docente Centro , Madrid , Spain
* Corresponding author.
Keywords: psychosis, myxedema, hypothyroidism, treatment

Conflict of interest:

No

Article ID: EPV1353

What is behind the silence? a case report of mutism.

Rodado-León B. 1 *
Bermejo Pastor A. 1
García Carpintero A. 2
Jiménez Cabañas M. 2
Alberdi Páramo Í. 3
1 Hospital Clínico San Carlos, Institute of Psychiatry And Mental Health , Madrid , Spain
2 Hospital Universitario Clínico San Carlos, Instituto De Psiquiatría Y Salud Mental , Madrid , Spain
3 Hospital Clínico San Carlos ., Institute of Psychiatry And Mental Health ., Madrid , Spain
* Corresponding author.
Keywords: mutism, persistent delusional disorder, Psychotic Symptoms

Conflict of interest:

No

Article ID: EPV1358

Deterioration of oral and dental health in people with schizophrenia treated with antipsychotic medication

Urien L. 1 *
Jauregizar N. 1
Rodriguez B. 2
Arnaiz A. 2
Pérez L. 2
Touzón R. 2
López-Vicente J. 3
Eguia Del Valle A. 3
Pacheco L. 2
Uriarte J.J. 2
Landaluce J.I. 2
Moreno M.C. 2
Garay M.A. 2
Morera-Herreras T. 1
1 University of the Basque Country , Pharmacology, Leioa , Spain
2 Osakidetza , Basque Health Service, Biocruces Bizkaia Health Research Institute, Bizkaia Mental Health Network , Bilbao , Spain
3 University of the Basque Country , Stomatology Ii, Leioa , Spain
* Corresponding author.
Keywords: schizophrenia, Dental, antipsychotic

Conflict of interest:

No

Article ID: EPV1396
Subjects: Abstract, Sexual Medicine and Mental Health

Am I hipersexual because I am bored?

Martins Ramos B. *
Barbosa D.
Delgado A.
Osório E.
São João Hospital and University Center , Psychiatry And Mental Health Department, Porto , Portugal
* Corresponding author.
Keywords: sexology, hipersexuality, Addiction, boredom

Conflict of interest:

No

Article ID: EPV1397

Ovotesticular disorder of sexual development and gender dysphoria – how far can we go?

Martins Ramos B. 1 *
Barbosa D. 2
Delgado A. 1
Osório E. 1
1 São João Hospital and University Center , Psychiatry And Mental Health Department, Porto , Portugal
2 Sao Joao Hospital and University Centre , Psychiatry, Porto , Portugal
* Corresponding author.
Keywords: gender dysphoria, ovotestis, sexology, medical intervention

Conflict of interest:

No

Article ID: EPV1435
Subjects: Abstract, Suicidology and Suicide Prevention

Passive and active suicidal ideation among psychiatry residents in spain: a cross-sectional study.

Hernández-Calle D. 1 *
Cano Arenas A. 1
Carracedo Sanchidrián D. 1
Herranz Hernández R. 2
Bravo-Ortiz M.F. 1
1 Hospital Universitario La Paz , Psychiatry, Madrid , Spain
2 Hospital Clínico San Carlos , Preventive Medicine, Madrid , Spain
* Corresponding author.
Keywords: Suicidal ideation, Medical Residents, Psychiatry residents

Conflict of interest:

No

Article ID: EPV1439

Oddly happy after suicide attempt

Santos Silva L. *
Almeida C.
Miranda J.
Barbosa M.
Duarte M.
Melim J.
Centro Hospitalar de Leiria, Serviço Psiquiatria E Saúde Mental , Leiria , Portugal
* Corresponding author.
Keywords: Suicide, Dépression, brain injury, Hypomania

Conflict of interest:

No

Article ID: EPV1441

Characterization of psychological pain in depressed patients

Alacreu Crespo A. 1 2 *
Guillaume S. 1 2
Courtet P. 1 2
Olie E. 1 2
1 INSERM, U1061: Neuropsychiatry: Epidemiological And Clinical Research , Montpellier , France
2 CHU Montpellier, Emergency Psychiatry And Acute Care , Montpellier , France
* Corresponding author.
Keywords: Suicide, Psychological pain, Dépression

Conflict of interest:

No

Article ID: EPV1464

Suicide attempts and self-harm behavior in day hospital patients

Rodríguez Muro E.
García-González P. *
Hospital de Día de Villaverde, Psychiatry, Madrid , Spain
* Corresponding author.
Keywords: Suicide, Self-harm, DAY HOSPITAL

Conflict of interest:

No

Article ID: EPV1476
Subjects: Abstract, Training in Psychiatry

Antipsychotics complications monitoring: evaluation of practice among young psychiatrists

Ennazk S. *
Abrache M.
Akebour K.
Adali I.
Manoudi F.
Asri F.
Equipe de recherche pour la santé mentale , service de psychiatrie, Hôpital Ibn Nafis, CHU MARRAKECH , Psychiatry, Marrakech , Morocco
* Corresponding author.
Keywords: complications, Young psychiatrists, antipsychotics, monitoring

Conflict of interest:

No

Article ID: EPV1490
Subjects: Abstract; Women, Gender and Mental Health

Be born woman, be trans, have a mental health diagnosis: when stigma puts your life at risk.

Fontecha Banegas L. 1 *
Cuevas I. 2
Moreno Alonso I. 1
1 Hospital Universitario Principe de Asturias , Psiquiatria, Alcala de Henares , Spain
2 Hospital Universitario Principe de Asturias , Psiquiatria, Alcalá de Henares , Spain
* Corresponding author.
Keywords: Stigma, mental health, organic screening, consequences

Conflict of interest:

No

Article ID: EPV1504

Use of oral aripiprazole in a pregnancy woman with schizophrenia. Case report.

Rodriguez De Lorenzo M. 1 *
Carrajo Garcia C. 2
Loeck De Lapuerta C. 3
Alonso Sánchez E. 2
Del Sol Calderón P. 4
1 Hospital Universitario Ramon y Cajal , Psychiatry, Madrid , Spain
2 HOSPITAL UNIVERSITARIO RAMON Y CAJAL , Psychiatry , MADRID , Spain
3 RAMON Y CAJAL UNIVERSITY HOSPITAL , Psychiatry , MADRID , Spain
4 HOSPITAL UNIVERSITARIO PUERTA DE HIERRO MAJADAHONDA , Psychiatry , MADRID , Spain
* Corresponding author.
Keywords: aripiprazole, schizophrenia, lactation, Pregnancy

Conflict of interest:

No

Article ID: EPV1505

Use of oral olanzapine in a pregnancy woman with bipolar disorder. Case report.

Rodriguez De Lorenzo M. 1 *
Loeck De Lapuerta C. 2
Alonso Sánchez E. 3
Carrajo Garcia C. 3
Del Sol Calderón P. 4
1 Hospital Universitario Ramon y Cajal , Psychiatry, Madrid , Spain
2 RAMON Y CAJAL UNIVERSITY HOSPITAL , Psychiatry , MADRID , Spain
3 HOSPITAL UNIVERSITARIO RAMON Y CAJAL , Psychiatry , MADRID , Spain
4 HOSPITAL UNIVERSITARIO PUERTA DE HIERRO MAJADAHONDA , Psychiatry , MADRID , Spain
* Corresponding author.
Keywords: breastfeeding, Bipolar disorder, olanzapine, Pregnancy

Conflict of interest:

No

Article ID: EPV1570
Subjects: Schizophrenia and Other Psychotic Disorders

Hospitalizations of patients with severe schizophrenia treated in a community based, case managed program vs. standard care. A ten-year follow-up

Diaz-Fernandez S. *
Fernandez-Miranda J.J.
Spain
* Corresponding author.
Keywords: schizophrenia, case management, treatment adherence, hospitalization

Article ID: S046
Subjects: Abstract, Symposium Immune alterations in early psychosis: new goals for diagnosis and treatment

Common genes in neurodevelopment and immune-inflammatory pathways impacted in schizophrenia?

Ramoz N. *
Gorwood P. *
INSSERM, Institute of Psychiatry and Neuroscience Of Paris , Paris , France
* Corresponding author.
Keywords: neurodevelopment, genetics, schizophrenia, psychosis

Conflict of interest:

No

Article ID: S098
Subjects: Abstract, Mental Health and the Transition from Child to Adult Mental Health Services in Europe

Improving transitions in europe: lessons from the milestone project

Tuomainen H. 1 *
Warwick J. 2
Singh S. 1
1 University of Warwick, Warwick Medical School , Mental Health and Wellbeing, Coventry , United Kingdom
2 University of Warwick, Warwick Medical School , Clinical Trials Unit, Coventry , United Kingdom
* Corresponding author.
Keywords: mental health services, young people, Transition, Europe

Conflict of interest:

No

Article ID: S168
Subjects: Abstract, Can innovative technologies optimise mental health care? Some shining examples

The smart-crisis project: ecological risk assessment in suicidal patients

Lopez-Castroman J. 1 2 3
1 Inserm, Unit 1061, Montpellier , France
2 University of Montpellier , Psychiatry, Montpellier , France
3 Nimes University Hospital , Psychiatry, Nimes, France
Keywords: digital phenotype, ecological momentary assessment, e-health, suicidal behavior

Conflict of interest:

No

Article ID: W006
Subjects: Abstract, Workshop Secondary prevention of suicide

The role of antidepressants in the secondary prevention of suicide

Lopez-Castroman J. 1 2 3
1 Nimes University Hospital , Psychiatry, Nimes, France
2 Inserm, Unit 1061, Montpellier , France
3 University of Montpellier , Psychiatry, Montpellier , France
Keywords: Antidepressant response, activation syndrome, adverse effects

Conflicts of Interest:

No

Article ID: W059
Subjects: Abstract, An Exciting Time for Research for Early Career Psychiatrists: Methods and Perspectives

Social media analysis for psychiatric research: mental health infodemiology in twitter

Alvarez-Mon M.
University Hospital Infanta Leonor , Department of Psychiatry, Madrid , Spain
Keywords: Infodemiology, Twitter, mental health

Conflicts of Interest:

No

Article ID: W074
Subjects: Abstract, The Complexity of Bipolar Disorders

Neuropsychological impairment in patients with bipolar disorder

Martinez-Aran A.
Clinical Institute of Neuroscience , Hospital Clinic, University of Barcelona, IDIBAPS, CIBERSAM, Bipolar and Depressive Unit, Barcelona , Spain
Keywords: Bipolar disorder, neurocognition, functioning

