
JOURNAL INFORMATION
==============================
NLM Title Abbreviation: Eur Psychiatry
Journal ID: Eur Psychiatry
NLM Journal ID: 9111820
Journal ID: EPA

European Psychiatry

ISSN: 0924-9338
EISSN: 1778-3585
Publisher: Cambridge University Press

ARTICLE INFORMATION
==============================
PMCID: PMC9412719
DOI: 10.1192/j.eurpsy.2021.10
Article ID: S0924933821000109
Article version: 1
Subjects: Corrigendum

EPV0798 - CORRIGENDUM

Publication date: 2020 Jul
Electronic publication date: 2020 Sep 4
Volume: 63
Issue: Suppl 1
Issue title: Abstracts of the 28th European Congress of Psychiatry
First page: S714
Last page: S714
Copyright: © The Author(s), 2021. Published by Cambridge University Press on behalf of the European Psychiatric Association 2021
Copyright year: 2021
Copyright holder: The Author(s), 2021. Published by Cambridge University Press on behalf of the European Psychiatric Association
License: This is an Open Access article, distributed under the terms of the Creative Commons Attribution licence (http://creativecommons.org/licenses/by/4.0/), which permits unrestricted re-use, distribution, and reproduction in any medium, provided the original work is properly cited.
License URL: https://creativecommons.org/licenses/by/4.0/

Erratum for: EPA 2020 Abstracts, Part 2 63 Suppl 1 8 12 2020 S629 S712 European Psychiatry 10.1192/j.eurpsy.2020.95 PMC9412732
Reference count: 1
Page count: 1

==============================
An author’s name was published incorrectly in the Part 2 section of the Abstracts of the 28th European Congress of Psychiatry supplement. The author’s name should be “L.V. Banerjee”, instead of “L. Banjee”. The supplement has since been updated to rectify the author name.

The authors apologise for this error.


Reference

(2020). EPA 2020 Abstracts, Part 2. European Psychiatry 63(S1), S629–S712. 10.1192/j.eurpsy.2020.95
