
JOURNAL INFORMATION
==============================
NLM Title Abbreviation: Eur Psychiatry
Journal ID: Eur Psychiatry
NLM Journal ID: 9111820
Journal ID: EPA

European Psychiatry

ISSN: 0924-9338
EISSN: 1778-3585
Publisher: Cambridge University Press

ARTICLE INFORMATION
==============================
PMCID: PMC9412711
DOI: 10.1192/j.eurpsy.2020.96
Article ID: S0924933820000966
Article version: 1
Subjects: Addendum

EPA 2020 Abstracts, Part 2 - ADDENDUM

Publication date: 2020 Jul
Electronic publication date: 2020 Dec 8
Volume: 63
Issue: Suppl 1
Issue title: Abstracts of the 28th European Congress of Psychiatry
First page: S713
Last page: S713
Copyright: © The Author(s) 2020
Copyright year: 2020
Copyright holder: The Author(s)
License: This is an Open Access article, distributed under the terms of the Creative Commons Attribution licence (http://creativecommons.org/licenses/by/4.0/), which permits unrestricted re-use, distribution, and reproduction in any medium, provided the original work is properly cited.
License URL: https://creativecommons.org/licenses/by/4.0/

Erratum for: EPA 2020 Abstracts, Part 2 63 Suppl 1 8 12 2020 S629 S712 European Psychiatry 10.1192/j.eurpsy.2020.95 PMC9412732
Reference count: 1
Page count: 1

==============================
In the 2020 supplement for the Abstracts of the 28th European Congress of Psychiatry, a number of qualifying abstracts were accidentally omitted. The supplement has since been updated to publish the omitted abstracts.

The Publisher apologies for this error.


Reference

(2020). EPA 2020 Abstracts, Part 2. European Psychiatry 63(S1), S629–S712. 10.1192/j.eurpsy.2020.95
